# Optimising DNA origami assembly by reducing off-target interactions

**DOI:** 10.1038/s41467-026-73387-4

**Published:** 2026-05-26

**Authors:** Ben Shirt-Ediss, Emanuela Torelli, Silvia Adriana Navarro, Hadeel Khamis, Ariel Kaplan, William Trewby, Juan Elezgaray, Nima Moradzadeh, Michael Haydell, Daniel Keppner, Michael Famulok, Kai Armstrong, Natalio Krasnogor

**Affiliations:** 1 Interdisciplinary Computing and Complex Biosystems (ICOS) Research Group, School of Computing, Newcastle University, Newcastle upon Tyne, UK; 2 Department of Agriculture, Food, Environmental and Animal Sciences, Università degli Studi di Udine, Udine, Italy; 3 Faculty of Biology and Russell Berrie Nanotechnology Institute, Technion—Israel Institute of Technology, Haifa, Israel; 4 Durham University, Durham, UK; 5 Centre de Recherche Paul Pascal, CNRS, U. Bordeaux, Pessac France; 6 LIMES Chemical Biology Unit, Universität Bonn, Bonn, Germany; 7 Present Address: London Centre for Nanotechnology, University College London (UCL), London, UK

**Keywords:** Nanostructures, DNA nanostructures, Computational science, Optical manipulation and tweezers

## Abstract

DNA origami enables the programmable self-assembly of nucleic acids into precisely defined nanostructures, yet the influence of primary base sequence on folding reliability remains incompletely understood. In particular, off-target interactions between scaffold and staple strands may introduce kinetic traps and reduce assembly yield, even when the intended Watson-Crick complementarity is preserved. Here we show that scaffold sequence strongly affects DNA origami assembly through the prevalence of off-target binding reactions implicit in the chosen base sequence. We developed a multi-objective computational framework that scores candidate scaffold sequences according to four classes of off-target interactions and selects variants predicted to minimise these effects for a given origami design. Using this approach, we identified both favourable and unfavourable scaffold regions from biological and synthetic sequences and tested them experimentally across 2D and 3D DNA origami structures. Atomic force microscopy showed that scaffolds predicted to have fewer off-target interactions consistently folded with higher yield, whereas off-target-prone scaffolds largely failed despite having fully complementary staple sets. Single-molecule optical tweezers further revealed that scaffold variants with fewer predicted off-target interactions assemble into more mechanically uniform origami structures. These results establish off-target sequence effects as a major determinant of origami folding and we provide a software tool to select scaffold sequences that minimise off-target reactions for any DNA origami design.

## Abstract (web summary)

Off-target binding can result in kinetic traps reducing the efficiency of DNA origami assembly. Here, the authors develop a software tool to design scaffold & staples with chosen sequences to avoid unwanted molecular interactions, yielding stronger, more uniform nanostructures.

## Introduction

The scaffolded DNA (and RNA) origami technique permits fabrication of nano-scale objects at high spatial precision via the programmable self-assembly of nucleic acids^1^. The bio-compatibility and bio-interpretability of origami nano-structures naturally position them for future biomedical applications ^2–4^.

Rather than top-down fabrication, DNA origami nanostructures are obtained through a complex ‘bottom-up’ self-assembly reaction involving hundreds of different types of single-stranded nucleic acids interacting in parallel via hybridisation, melting and strand-displacement reactions in a decreasing temperature ramp applied over an extended period. The result of the cooled self-assembly reaction is, ideally, the target nanostructure at high copy number and yield.

An increasing number of experimental and theoretical/computational studies are being devoted to understanding the detailed biophysics of origami self-assembly^5^, both to pinpoint which factors are the most important to achieve high-yield origami folding and to understand the surprising robustness of the folding process.

These works have investigated how in vitro origami assembly is affected by factors such as the origami design itself^6–10^ (i.e., the staple and scaffold routing patterns), the temperature protocol^11,12^, the stoichiometric ratio of staples to scaffold^10,13,14^, the type and amount of salts in the assembly buffer^12,15^, the solvent^16^ and the primary base sequences of the scaffold and staple strands^7^.

The focus of the current study is on how the latter factor of base sequence can be manipulated to increase origami self-assembly yield.

Although base sequences encode the folding information itself and have a combinatorially large space of possibilities, this assembly factor has often only been explored coarsely. For example, higher GC content scaffolds have been shown to assemble at lower salt concentrations^17^ and higher nearest-neighbour base energies have been experimentally demonstrated to generally raise the melting temperature of the structure^17,18^.

However, more nuanced effects of base sequence on self-assembly are emerging. For example, in a recent study, scaffold sequence has been demonstrated to be able to control the folding pathway of specialised origami designs that have two distinct possible end states^19^ by specifying the locations of folding nucleation points at the early high-temperature phase of folding.

Another nuanced but potentially important effect of the base sequence is the long-range effects of off-target side reactions (i.e., partial mis-bindings) that particular sequences make possible throughout the whole system of hundreds of assembling strands. This was first touched on by Rothemund^20^, who avoided base positions 5515-5587 of the M13 scaffold in his original origami designs because it contained a strong hairpin loop. Since then, some origami CAD tools have included design algorithms to make synthetic scaffold sequences deficient in repeated regions and with controlled GC content^21^. In a similar vein, 4-letter de Bruijn sequences^22^ have also been suggested as suitable synthetic scaffold sequences due to their ability to repress all repeats shorter than a certain number of bases, thus, in principle, providing ‘unique addressability’ for binding staples^23^^,^^17^^,^^24^.

Overall, however, off-target reactions in DNA origami self-assembly have so far been largely ignored by the DNA nanotechnology community. This is perhaps because origami assembly has been conceived as a ‘sequence-agnostic nucleation and growth system’^14^. As observed by atomic force microscopy (AFM)^12,25^, it likely involves the formation of nucleation centres at high temperature where on-target staples bind and make short loops in the scaffold strand. Bound staples then begin to ‘organise the scaffold’^20^ and co-operatively help the binding of immediately adjacent on-target staples, therefore enabling efficient folding fronts to rapidly extend from the initial nucleation sites. Folding from high temperature also helps to ensure that on-target staple bindings form before weaker off-target bindings have any opportunity, and, if off-target bindings are present, they are assumed to be corrected by strand displacement from invading on-target staples^20^.

Nevertheless, as robust as self-assembly described in this way seems, it is conceivable that the nucleation-and-growth process could still get kinetically trapped (for potentially long times) away from the target equilibrium origami structure if base sequences made unfortunate off-target bindings possible.

In the limit case, it could be agreed that a scaffold sequence made of a short repeating sequence (e.g., ACGT) would certainly not have a viable nucleation-and-growth self-assembly process when mixed with its complementary staple set. However, the key question is whether more heterogeneous scaffold sequences could still have assembly problems. It is conceivable that heterogeneous scaffold sequences could sometimes contain problematic regions of strong secondary structure that could act to block on-target staple binding sites. The ability of on-target staples to strand-displace mis-bound configurations could be hampered by factors like the scaffold having a high effective concentration with respect to itself, or off-target staples (in excess) reversibly binding and occluding toeholds. Furthermore, staples with a leg misbound could conceivably permit staple blocking^14,26^ by other copies of the same staple. Finally, off-target bindings between the staples themselves could slow the kinetic rate of on-target staple attachment to the scaffold.

Off-target reactions could be considered as more relevant when scaffold sequences are derived from repeat-prone biological sources^27^, when staple legs are fairly short (7 or 8nt) and bind initially at intermediate temperatures, or when constant-temperature folding with no initial thermal denaturation step is employed^12,28,29^.

Definitive answers about when the ‘noise’ of off-target reactions is sufficient to derail the ‘signal’ of the nucleation-and-growth assembly process for specific origami designs with specific scaffold/staple sequences could seemingly be obtained from theoretical/computational models of origami folding.

Simulation of DNA origami folding by coarse-grain molecular dynamics^26^ does, in principle, rigorously include all off-target reaction possibilities because of the base-level resolution. However, such simulations are practically restricted to second timescales after months of computation and typically require artificial assembly conditions (a small origami, high staple concentrations, relatively high constant temperature) to be feasible. Further coarse-graining to 8nt per bead and using a switchable force field^8^ has more recently allowed self-assembly for kilobase-size origamis to be simulated (again at second timescales, constant temperature). However, the latter model only considers hybridising bead pairs to be those in the target origami design; accordingly, kinetic traps are limited to situations where on-target binding sites become sterically inaccessible during folding. Moreover, an 8nt bead resolution cannot incorporate strand-displacement reactions – a crucial mechanism for resolving off-target binding sites.

Approximate ‘domain-level’ kinetic models of origami assembly based on mass action rates between hybridisation states^30^^,^^6^^,^^18^ can access experimentally relevant timescales (hours) by sacrificing exact geometric information for a purely topological hybridisation state. Moreover, they are able to include a full-temperature annealing ramp in the simulation. However, again, current models only consider on-target staple-scaffold bindings in their transition possibilities, leaving staple blocking (at very high staple concentrations) as the only mechanism able to create kinetic traps. While off-target reactions and associated strand displacement mechanisms could be (partially) included in these mass action models in future by relaxing the domain-level constraint, it is a formidable undertaking to implement: accurate reaction enumeration and parameterisation of possible hybridisation and (non-standard) strand-displacement reactions is non-trivial, and difficult computational problems such as stiff dynamics and efficient shortest path calculation on complex graphs must be overcome.

Furthermore, all theoretical models mentioned above only track a single origami instance and do not take into account, e.g., possible undesired inter-origami binding effects, like hybridisations between independent scaffold strands in solution.

Given the challenges of rigorously incorporating off-target side reactions into ‘dynamic simulation’ folding models, in this work, we take an alternative ‘static optimisation’ approach to identifying origami scaffold sequences that are likely to fold reliably for a particular origami design.

Our main hypothesis is that scaffold sequences (and complementary staple sets) that lead to a reduced number of off-target reactions are statistically less likely to get kinetically trapped during the origami self-assembly process.

We present a computational method based on exactly enumerating and minimising four types of off-target side reactions for a particular origami design. Our method uses multi-objective optimisation techniques (for example, also recently applied to RNAs^31^, proteins^32^ and gene circuits^33^) to identify a ‘Pareto set’ of scaffold sequences that are best able to minimise off-target reactions from a large pool of alternatives.

In the remainder of the paper, we describe our computational method and demonstrate in vitro, using as targets a DNA origami triangle, rectangle, 6-helix bundle (6-HB) and a tetrahedron, that selecting scaffold sequences with a high number of associated off-target reactions leads to failed origami folding (even when all staples are exact Watson-Crick complements to the scaffold). Conversely, we demonstrate scaffold sequences with fewer off-target reactions assemble with a higher yield in all origamis. Besides AFM verification of our computational predictions, we also conduct detailed single-molecule optical tweezer (OT) experiments on the triangle origami design, with results that align with and further confirm our calculations.

## Results

### Principles of multi-objective scaffold sequence selection

**Fig. 1:**
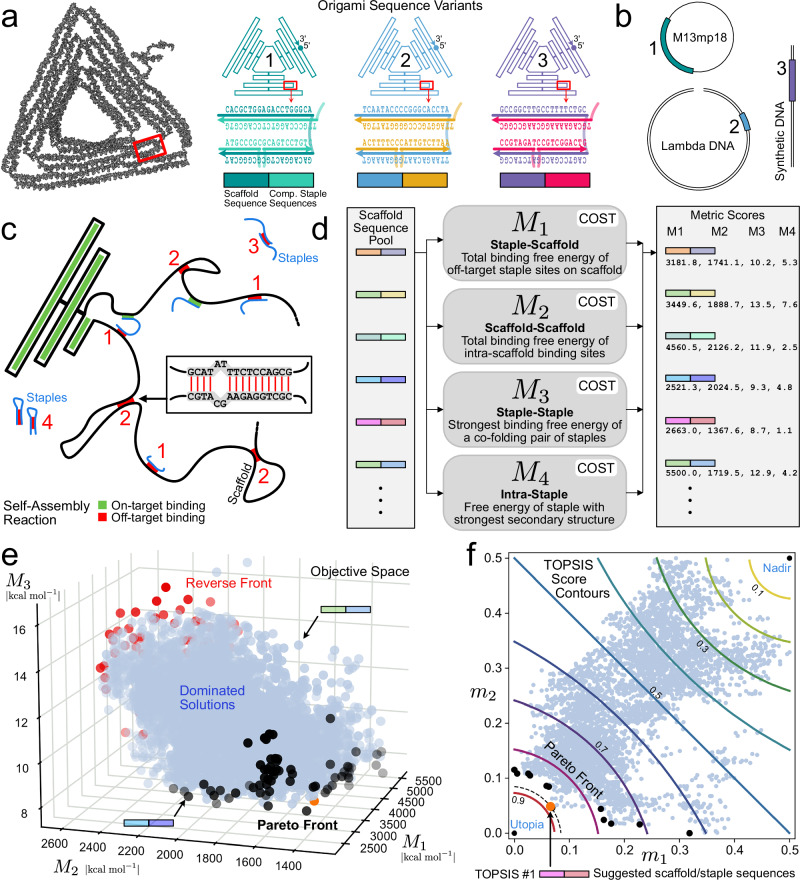
Principles of multi-objective scaffold sequence selection. **a** The same DNA origami target design can be realised by many different scaffold sequences. Three possible sequence variants are displayed for a triangle origami (rendered by oxDNA.org^50^). Each variant, denoted as a pair of coloured blocks, consists of a unique scaffold sequence along with the Watson-Crick complementary staple sequences that pin the scaffold strand into a specific rotation. **b** Scaffold sequences are commonly sourced from regions of biological vectors, custom-cloned sequences or synthetic sequences. **c** The origami self-assembly reaction involves numerous off-target bindings (red) in addition to the intended on-target staple-scaffold bindings (green). Off-target bindings are classified into four types 1-4, which in turn are measured by metrics *M*_1_ ⋯ *M*_4_. **d** Scaffold sequence selection takes place by scoring a large pool of scaffold/staple sequence variants on four cost criteria that quantify the extent of each off-target binding site type in panel (c). Full definition of each metric in Supplementary Note 2. **e** After scoring, all variants (here *n* = 5000) are mapped to a low-dimensional objective space (*M*_4_ scores are omitted for 3D visualisation). The Pareto front of optimal trade-off variants is computed in this space (black dots). **f** A multi-criteria decision-making method (MCDM; here TOPSIS) ranks Pareto candidates and aids a human decision maker in choosing a single scaffold/staple sequence variant to order for in vitro assembly. To visualise TOPSIS ranking, TOPSIS score contours are shown on a 2-dimensional objective space: this method works similarly on a 3 or 4-dimensional objective space. See Supplementary Note 3.

Our multi-objective approach to selecting reliable folder origami scaffold sequences is summarised in Fig. 1. A target origami design is specified a priori and a large pool of ‘sequence variants’ (potential scaffold sequences, each with their Watson-Crick complementary staples) is scored on four thermodynamic objective functions, which we also call ‘metrics’. Each of these metrics *M*_1_ to *M*_4_ characterise the prevalence of a particular class of off-target binding site during folding. Metric *M*_1_ represents the total energy of staple-scaffold off-target binding sites; *M*_2_ the total energy of scaffold-scaffold binding sites (all off-target); *M*_3_ the energy of the worst staple-staple co-fold, and *M*_4_ the energy of the worst staple hairpin (see Supplementary Note 2 for detailed definitions). When an objective function is minimised to zero, it signifies that off-target bindings of that particular type do not exist.

Rather than making a direct prediction of origami assembly yield for a sequence variant, the premise of our method is instead to calculate four scores that are *general heuristics* for high-yield folding (but not absolute measures of it). Our hypothesis is that sequence variants with low-scoring objectives are statistically more likely to fold with high yield than sequence variants with high-scoring objectives because fewer off-target sites correlate with reduced total kinetic traps on the origami folding pathway. That is, low scores amongst (most) metrics help identify reliable folders.

The relative comparison of sequence variants takes place in objective space (Fig. 1e) where a set (called the ‘Pareto front’) of optimal trade-off sequence variants becomes defined (black dots, see Supplementary Note 3). Sequence variants on the Pareto front are special in that they represent the set of optimal trade-offs for the optimisation problem: for Pareto variants, it is not possible to decrease the prevalence of one type of off-target reaction without increasing the prevalence of other types of off-target reaction. A Pareto front exists because metrics are antagonistic and it is typically impossible for a sequence variant to minimise all metrics *M*_1_ to *M*_4_ to zero simultaneously. Similarly, a reverse Pareto front of ‘worse’ variants can be defined (red dots, Fig. 1e) by treating the scoring metrics as benefits to be maximised rather than costs to be minimised.

For a given origami design and pool of scaffold/staple sequence variants realising that design, our method returns the Pareto set of sequence variants with the best trade-offs at minimising off-target reactions during self-assembly. Therefore, not one sequence variant, but a cloud of potentially good sequence variants is returned. Using all four scoring metrics, the Pareto set is typically 2–3% of the initial pool of sequence variants. When the initial sequence variant pool has a size of 5000, the Pareto set is over 100 variants. The number of Pareto sequence variants is still too many for a human decision-maker to manually review and choose between. Therefore, for the last step, we use a Multi-Criteria Decision Making (MCDM) method^34^ to help decide which single variant on the Pareto front is the most appropriate scaffold/staple sequence set to order for implementing the origami design. An MCDM method provides a mathematical framework to rank the trade-off variants on the Pareto front, allowing the identification of a single ‘best compromise’ solution, for example, by calculating which variant is geometrically closest to a theoretical ‘utopia point’ where all metrics are minimised. See Supplementary Note 3 for definitions of the SAW, KNEE and TOPSIS MCDM methods used. For each of the MCDM methods, in the absence of better information, we weighted all of the thermodynamic scoring metrics *M*_1_ to *M*_4_ equally.

**Fig. 2:**
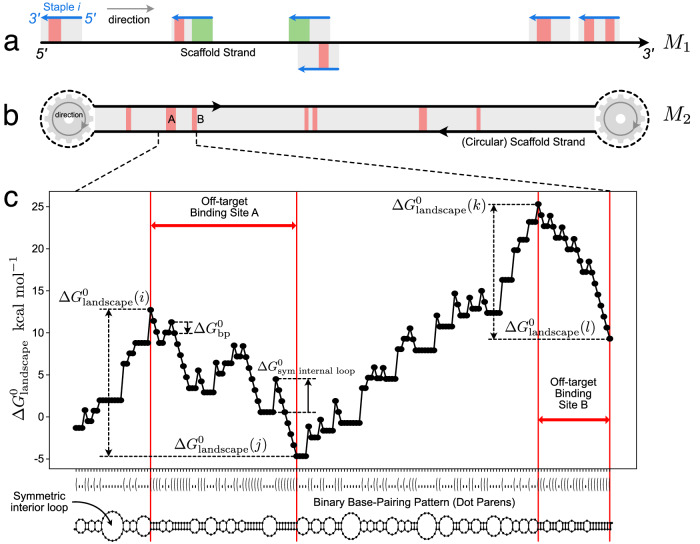
Hybridisation energy model to detect off-target binding sites. Metrics *M*_1_ and *M*_2_ use a fast sequence-averaged energy model of hybridisation to detect staple-scaffold and scaffold-scaffold binding sites, respectively. The energy model includes the possibility of symmetric interior loops in binding sites between two nucleic acid strands. **a** In Metric *M*_1_, each staple is moved anti-parallel to the scaffold strand in single base increments and the binary base-pairing pattern (`dot parens') is calculated at each position from the sequence alignment of the two strands. This binary base-pairing pattern is converted to an approximate hybridisation energy landscape (see panel (c)), which detects both on-target (green) and potential off-target (red) binding sites. **b** In Metric *M*_2_, the scaffold strand is aligned against itself to detect scaffold-scaffold binding sites (all of which are off-target). For circular scaffolds, the procedure to find off-target hybridising regions is approximately analogous to stretching the scaffold strand like a continuous band between two rollers; the hybridisation energy landscape of the central `helix' region is calculated for each single base rotation of the rollers. (Note, this does not mean the scaffold strand is actually being rotated through the origami structure). A section of this energy landscape is shown in panel (**c**). **c** A hybridisation energy landscape is calculated by decrementing the free energy by $\Delta {G}_{{{{\rm{bp}}}}}^{0}$ for each base pair encountered and incurring a free energy penalty of $\Delta {G}_{{{{\rm{syminteriorloop}}}}}^{0}$ (dependent on loop length) for each symmetric interior loop encountered. A binding site (either on- or off-target) is detected when the difference in free energy between two points on the landscape *i* and *j* is $\Delta {G}_{{{{\rm{site}}}}}^{0}=\Delta {G}_{{{{\rm{landscape}}}}}^{0}(j)-\Delta {G}_{{{{\rm{landscape}}}}}^{0}(i)+(\Delta {G}_{{{{\rm{assoc}}}}}^{0}+\Delta {G}_{{{{\rm{bp}}}}}^{0})\le -7.0{{{{\rm{kcalmol}}}}}^{-1}$ at 37^∘^C. The free energy values are parameterised using NUPACK. See Supplementary Note 1 for details.

Finally, we developed a fast approximated energy model parameterised on NUPACK^35^ for calculating metrics *M*_1_ and *M*_2_ (summarised in Fig. 2). The energy model uses sliding window techniques to efficiently detect off-target binding sites between staples and scaffold and to detect regions where the scaffold can bind to itself. Binding sites are detected at base-level resolution and the energy model allows for mismatching symmetric interior loops of different sizes within binding sites (see Supplementary Note 1). Of note, the energy model takes into account all potential scaffold-scaffold bindings when calculating *M*_2_, not just those bindings present in the minimum free energy (MFE) configuration of the scaffold strand. Also, it is much faster to calculate than the MFE configuration. For metrics *M*_3_ and *M*_4_ requiring fewer calculations, we call NUPACK to calculate binding energies based on full sequence information.

### There is significant scope for optimising scaffold sequences, especially those arising from biological vectors

To determine in which scenarios scaffold sequence selection is most effective at minimising off-target reactions, we first performed a large-scale computational investigation that used our method to select scaffold sequences for 14 different 2D and 3D origami designs (Supplementary Notes 4 and 5).

Each origami design had sequence variants (scaffold and complementary staple sequences) selected from three qualitatively different sequence pools; (i) a biological vectors pool ‘VEC’ containing 5000 scaffold sequences taken from different random contiguous regions of biological vectors pUC19 (2686bp), M13mp18 (7249nt), p7560, p8064 and Lambda DNA (48502bp); (ii) A random pool containing 5000 synthetic random 4-letter scaffold sequences, where each base had an occurrence probability of 0.25; (iii) A de Bruijn pool ‘DBS’ containing 5000 synthetic de Bruijn sequences. A de Bruijn sequence of order *k* has the special mathematical property that a window of length *k* bases (or larger) will never frame the same sequence fragment twice when it is moved along the sequence base-by-base^23^. We used *k* = 5 for scaffolds 257 to 1024nt; *k* = 6 for scaffolds 1025 to 4096nt, and *k* = 7 for scaffolds 4097nt to 16384nt. Note that the three sequence pools described above were different for each origami. Each sequence pool only provided a very sparse sampling of the combinatorially vast sequence space; however, this sparse sampling was already sufficient to create significant variation in the metric *M*_1_ to *M*_4_ scores.

Overall, our initial computational investigation revealed that the biological vectors pool VEC produced the largest variance in all scoring metrics *M*_1_ to *M*_4_ for each of the 14 test origamis. This is consistent with biological sequences typically having a non-uniform spatial distribution of GC content, which creates higher densities of repeated sub-sequences in specific regions.

Selection of scaffolds for all 14 test origamis from the VEC pool above yielded objective spaces where the Pareto and reverse fronts of sequence variants were separated by a large margin. Even for larger 8knt scaffold origamis in the test set, there was still an  ≈ 40% relative reduction in off-target sites from the worst sequence variant on the reverse front (with many off-target reactions) to the best sequence variant on the Pareto front (with fewest off-target reactions). Therefore, we reasoned that scaffold sequences derived from biological vectors are not only the most commonly used to fabricate origami, but also the most effective target for our multi-objective selection method.

For the synthetic sequence pools, we found that de Bruijn sequences had the best absolute performance across the test set of 14 origamis (Supplementary Note 5). These sequences optimally minimised the total number of off-target reactions between staples and scaffold (but we noted that *M*_1_ scores did not reduce to zero, since the energy model also acknowledges symmetric mismatches in binding sites that the de Bruijn sequence property does not protect against).

De Bruijn sequences also significantly decreased scaffold-scaffold bindings (*M*_2_). Interestingly, even though the formal de Bruijn sequence property^22^ was not originally developed in relation to nucleic acids – and hence did not consider base-pairing between a de Bruijn sequence and itself – this type of self-interaction also turns out to be quite minimal for de Bruijn sequences.

We found (synthetic) random sequences to minimise scaffold-scaffold interactions equally as well as de Bruijn sequences and to typically perform better than biological sequences (but worse than de Bruijn sequences) at minimising off-target staple-scaffold interactions (*M*_1_). This performance can be attributed to the fact that random scaffolds are unlikely to contain repeated or complementary segments.

In general, we observed little benefit in performing DNA origami scaffold selection from pools of de Bruijn or random sequences because of their generally good baseline performance (but see Supplementary Note 5 for more detail). In particular, de Bruijn sequences *by design*^23^ already incorporated some of the considerations we used in metric *M*_1_, and as mentioned above, also performed well on *M*_2_.

Additionally, we tried selecting scaffold rotations of the published scaffold sequence for those origamis in the test set that had a circular scaffold (where ‘scaffold rotation’ signifies the physical translation of the fixed-sequence scaffold strand around the scaffold routing path in the origami nanostructure by choice of a different set of complementary staples). We observed that rotating the scaffold strand did little to minimise the already present off-target reactions, particularly for larger origamis (Supplementary Note 5). This effect can be anticipated, as rotations of the scaffold only change the staple sequences and staples inherit similar shifting sequence patterns as the scaffold is rotated.

More generally, measuring the Pearson correlation between all unique pairs of metric scores *M*_1_ to *M*_4_ for all 14 origamis across all sequence pools, we detected no intrinsic linear correlation (Supplementary Note 6) between these metrics. Visual observation of scatter plots also revealed no non-linear correlations. This signified that the scoring metrics were orthogonal (derived independent information about off-target interactions).

Finally, we found that the particular MCDM method (SAW, KNEE or TOPSIS) used to select a single origami sequence variant from the Pareto front only made a difference in isolated cases. Largely due to the data normalisation scheme we used (Supplementary Note 3), the MCDM methods approximately agreed about the best candidate sequence variants on the Pareto front and the worst candidate variants on the reverse front in objective space.

### Selection of 2D and 3D DNA origami variants for in vitro assembly

**Fig. 3:**
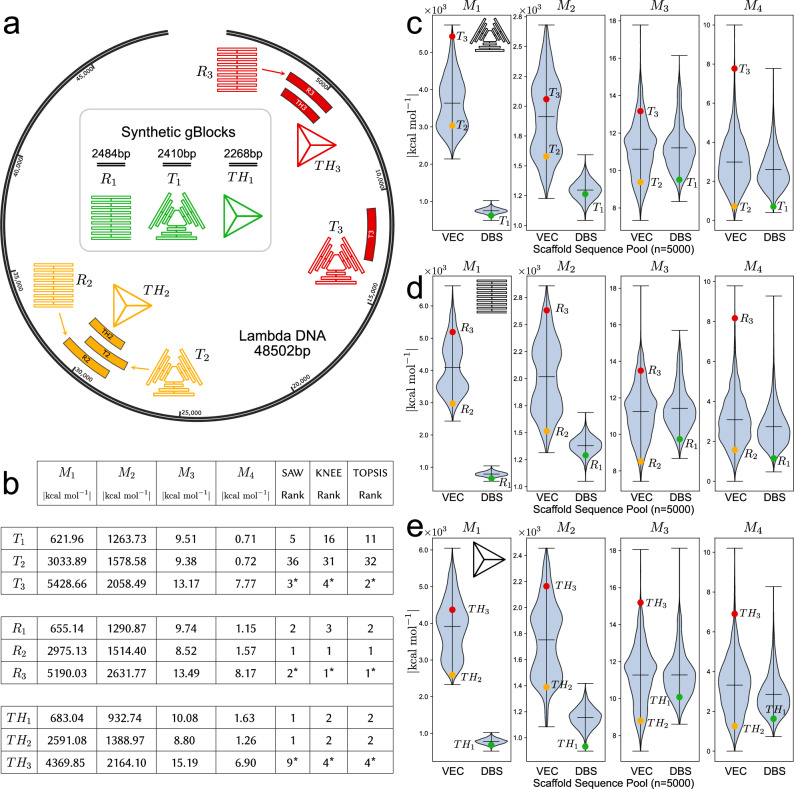
Triangle, rectangle and tetrahedron DNA origami sequence variants selected for in vitro assembly. Three variants of a DNA origami triangle *T*_1_, *T*_2_, *T*_3_ (2410nt scaffold), a DNA origami rectangle *R*_1_, *R*_2_, *R*_3_ (2484nt scaffold) and a 3D DNA origami tetrahedron *TH*_1_, *TH*_2_, *TH*_3_ (2268nt scaffold) were selected. **a** Lambda DNA sequence showing regions where `high off-target reaction' (*T*_3_, *R*_3_, *TH*_3_ in red) and `low off-target reaction' (*T*_2_, *R*_2_, *TH*_2_ in orange) biological scaffold sequences were obtained, according to scoring metrics *M*_1_ to *M*_4_. Each shape also had a `very low off-target reaction' synthetic de Bruijn scaffold sequence selected for comparison (*T*_1_, *R*_1_, *TH*_1_ in green). **b** Table of metric scores for all origami variants. Lower metric scores indicate fewer off-target binding sites during self-assembly. MCDM rankings were used as a guide to select variants from the Pareto and reverse fronts. Star * denotes rank on reverse front. **c** Full distributions of all scoring metrics for the triangle origami, over the VEC and DBS sequence pools, shown as violin density plots (*n* = 5000; error bars show min and max range and mean). The location of *T*_2_ and *T*_3_ in the VEC sequence pool distribution, and the location of *T*_1_ in the DBS sequence pool distribution, are marked. **d** Metric distributions for rectangle origami shown as violin density plots (*n* = 5000; error bars show min and max range and mean). The location of *R*_2_ and *R*_3_ in the VEC sequence pool distribution, and the location of *R*_1_ in the DBS sequence pool distribution, are marked. **e** Metric distributions for 3D tetrahedron origami shown as violin density plots (*n* = 5000; error bars show min and max range and mean). The location of *TH*_2_ and *TH*_3_ in the VEC sequence pool distribution, and the location of *TH*_1_ in the DBS sequence pool distribution, are marked.

To empirically test if selecting scaffold sequences from regions of biological sequences with relatively many or relatively few off-target reactions had a measurable effect on DNA origami assembly yield, we selected three sequence variants of each of the following shapes; a 2410nt 2D triangle^36^, a 2484nt 2D rectangle^23^, a 2268nt 3D tetrahedron (designed in ATHENA^37^) and a 1653nt 3D 6-helix bundle (designed manually) for in vitro assembly. See Fig. 3 for details and Supplementary Note 16 for the 6-helix bundle.

Triangle variants *T*_1_, *T*_2_ and *T*_3_ had a successively increasing number of potential off-target bindings. Variant *T*_1_ acted as a ‘control’, i.e., best predicted variant, with a synthetic scaffold sequence selected from a pool of 5000 de Bruijn sequences. Variant *T*_2_ was selected from the Pareto front of a pool of 5000 biological vector sequences (derived from contiguous fragments of the pUC19, M13mp18, p7560, p8064 and Lambda DNA sequences), while variant *T*_3_ was selected from the reverse front of the latter pool. Rectangle *R*_*n*_, tetrahedron *TH*_*n*_ and 6-helix bundle 6HB_*n*_ variants were obtained similarly.

It should be noted that all sequence variants of each origami shape had a fully complementary staple set to the scaffold sequence, and all made the target shape when fully self-assembled (design schematics in Supplementary Notes 8–11). The only factor differentiating variants was the number of off-target bindings possible in each case.

While the 5000 biological vector sequences prepared for each origami came from fragments of five different biological sequences, the scaffold sequences actually selected from the Pareto (*T*_2_,*R*_2_,*TH*_2_,6HB_2_) and reverse fronts (*T*_3_,*R*_3_,*TH*_3_,6HB_3_) all came from the Lambda DNA phage sequence (Fig. 3a).

### Synthesis of single-stranded DNA scaffold sequences

Twelve different dsDNA sequences were amplified and purified: the correct amplicon sizes were obtained as shown in Supplementary Note 13.

Next, the purified PCR products were selectively digested by T7 exonuclease overnight, purified to remove the exonuclease, concentrated and then the ssDNA was resuspended in water, discharging undesired ions. The resulting ssDNA sequences of lengths 2410 nt (triangle scaffolds), 2484 nt (rectangle scaffolds) and 2268 nt (tetrahedron scaffolds) showed higher gel mobilities compared to the non-digested dsDNA, as reported in Supplementary Note 13. A faint higher band was also observed in each scaffold variant and was considered as non-digested dsDNA.

To initially verify the T7 exonuclease-based digestion and the correct composition of triangle and rectangle ssDNA scaffolds, Sanger sequencing was considered. The results underlined no off-target degradation of sense-DNA as previously shown^38^. For this reason, the same protocol was applied to synthesise the 3D DNA origami scaffolds.

### Triangle and rectangle DNA origami variants: folding and pre-screening by gel electrophoresis

The origami self-assembly reactions were run in tris-acetate-EDTA (TAE) buffer containing 12.5 mM of magnesium acetate and with a 10-fold excess of staple strands, a common protocol for 2D origami folding^1,20^.

For each origami variant, 4 different thermal annealing protocols were considered. The mixtures were heated at 95 °C for a short period of time and then gradually cooled down to 20 °C following a slow (5h 40 min) or a fast (1h 15min) temperature ramp. Isothermal folding protocols were also considered, testing constant annealing temperature (37 °C) with or without initial denaturation at 95 °C.

To identify potential nanostructure side products (partially assembled and/or mis-folded intermediates) and aggregates, folding solutions incubated at different temperature conditions were first analysed by agarose gel electrophoresis (AGE), a method commonly used to initially assess origami assembly performance. The migration distances, the presence of smearing or multiple bands, and band sharpness were considered indicators of the folding quality, allowing us to select a specific folding condition for subsequent purification and characterisation by atomic force microscopy (AFM). Scaffold, staple strands, and scaffold mixed with non-complementary staples set were considered as negative controls. The formation of DNA origami nanostructures should lead to a mobility shift compared to the scaffold band, which is expected to disappear, while a scaffold mixed with a non-complementary staples set should migrate as the scaffold alone.

The Triangle DNA origami variants *T*_1_, *T*_2_ and *T*_3_ folded in isothermal conditions without initial denaturation showed similar band patterns characterised by a main band, a second fainter and diffuse band, and a smearing (Supplementary Fig. 32a–c, lane 7). While *T*_1_ and *T*_2_ variants folded in isothermal conditions with initial denaturation were characterised by similar band patterns and migration distance (Supplementary Fig. 32a, c, lane 8), the *T*_3_ variant showed aggregates visible in the loading well as a non-migrating band (Supplementary Fig. 32b, lane 8).

Gel electrophoretic analysis of the same variants folded following a fast ramp revealed different band patterns compared to the profiles reported above. In detail, sharp leading bands without intense smearing were visible (Supplementary Fig. 32a–c, lane 9), while aggregates were still formed in the *T*_3_ variant (Supplementary Fig. 32b, lane 9). The scaffold mixed with a non-complementary staple set showed the same migration distance as the scaffold alone (Supplementary Fig. 33b–c, lane S).

After 4 days (slow ramp) or 5 days (isothermal folding and fast ramp) at 4 °C, the structural stability of the variant assemblies was evaluated. *T*_1_ and *T*_2_ variants showed a similar band intensity (Supplementary Fig. 34a, c, lane 11) as on day 1 (Supplementary Fig. 32a–c, lane 9), indicating higher stability when compared to the *T*_3_ sample (fast ramp) and other origami samples folded at different temperature conditions.

Based on these results and considering the smearing and fainter bands noticeable in samples folded through a slow temperature ramp (Supplementary Fig. 33a–c, lane 12), we selected the fast ramp for further purification and characterisation by AFM (see below).

As in the triangle DNA origami gel image analysis, rectangle variants *R*_1_, *R*_2_, *R*_3_ folded in isothermal conditions without initial denaturation showed a similar band pattern characterised by a main band, a less intense second band and smearing (Supplementary Fig. 35a–c, lane 7). With initial denaturation, *R*_1_ and *R*_2_ variants were characterised by the same band pattern and migration distance with less visible smearing (Supplementary Fig. 35a, c, lane 8), while the *R*_3_ variant showed aggregates in the loading well, wide smearing and a main band migrating slightly slower (Supplementary Fig. 35b, lane 8).

*R*_1_, *R*_2_ and *R*_3_ samples annealed with a fast ramp had a similar electrophoretic behaviour when compared with both isothermal processes, but with a less pronounced smearing and slightly different migration distances (Supplementary Fig. 35a–c, lane 9). The negative controls, i.e., scaffold alone and scaffold with non-complementary staple set, showed the same migration distances between them (Supplementary Fig. 36a–c, lane S).

Considering the above results and the fainter bands corresponding to the slower ramp (Supplementary Fig. 36a–c, lane 12), assemblies obtained from the fast ramp were selected, purified, and imaged by AFM (see below). As noted for triangle variants, the rectangle assemblies showed different structural stability when stored at 4 °C: after 4 to 5 days, *R*_1_ and *R*_2_ samples (fast ramp) were more stable (Supplementary Fig. 37a, c, lane 11) compared to the scaffold and samples folded with a slow temperature ramp (Supplementary Fig. 37a–c, lane 12), while the *R*_3_ sample (fast ramp) band disappeared (Supplementary Fig. 37b, lane 11), underlying the assembly instability. The *R*_1_, *R*_2_ and *R*_3_ samples folded in isothermal conditions were relatively stable (Supplementary Fig. 37a–c, lanes 9 and 10).

### 2D triangle & rectangle and 3D tetrahedron DNA origami variants: purification and AFM imaging

Triangle, rectangle and tetrahedron DNA assemblies from a fast ramp annealing were purified by centrifugal filtration to remove the low molecular weight excess of staple strands and to concentrate the samples.

**Fig. 4:**
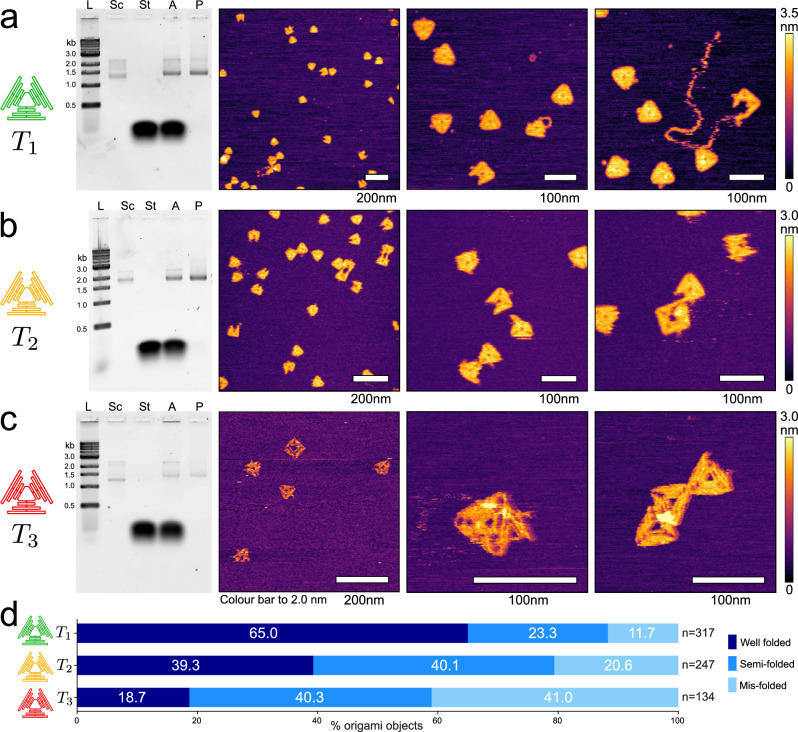
Triangle DNA origami sequence variants: agarose gel and AFM images. **a** Triangle variant *T*_1_. **b** Triangle variant *T*_2_. **c** Triangle variant *T*_3_. In (**a**), (**b**) and (**c**) the first panel shows laser scanned image of SYBR®Gold-stained 1% TBE agarose gel with lanes: L = 1kb Ladder; Sc = scaffold only; St = staples only; A = Origami self-assembly reaction mix (scaffold + staples); P = Purified assembly reaction mix used for AFM imaging. The assembly reaction was performed under a fast temperature ramp of 95 °C to 20 °C in 1h 15min (−0.1 °C per cycle, each cycle 6 seconds). All gel electrophoresis experiments were repeated 3 times. Source data are provided as a Source Data file. The remaining panels show representative high-resolution AFM images of the purified DNA self-assembly sample (P). **d** Manual quantification of origami objects on AFM images into “well-folded", “semi-folded" and “mis-folded" categories. The percent of origami objects in each category is shown.

**Fig. 5:**
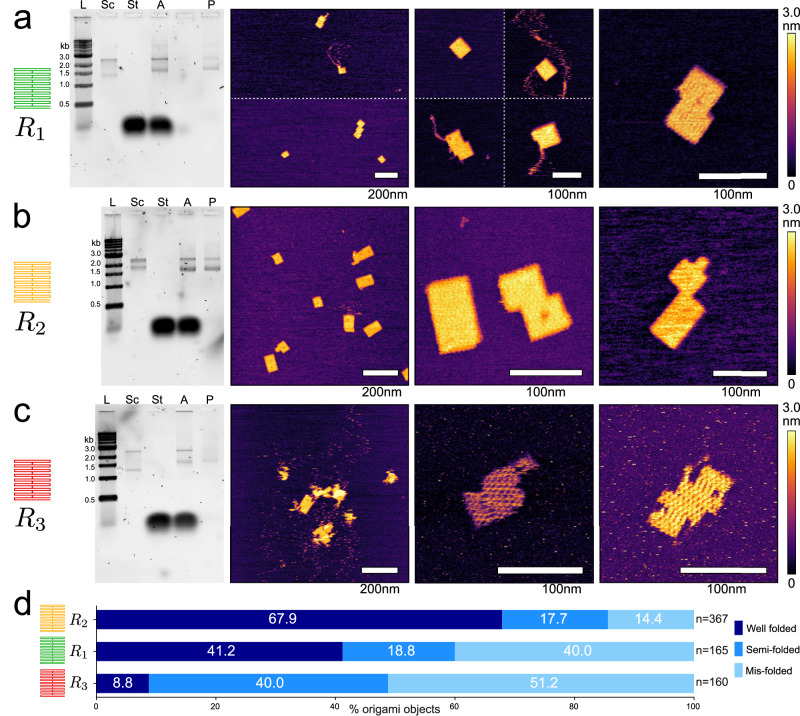
Rectangle DNA origami sequence variants: agarose gel and AFM images. **a** Rectangle variant *R*_1_. **b** Rectangle variant *R*_2_. **c** Rectangle variant *R*_3_. In (**a**), (**b**) and (**c**), the first panel shows the laser-scanned image of SYBR®Gold-stained 1% TBE agarose gel with lanes: L = 1kb Ladder; Sc = scaffold only; St = staples only; A = Origami self-assembly reaction mix (scaffold + staples); P = Purified assembly reaction mix used for AFM imaging. The assembly reaction was performed under a fast temperature ramp of 95 °C to 20 °C in 1h 15min (−0.1 °C per cycle, each cycle 6 seconds). All gel electrophoresis experiments were repeated 3 times. Source data are provided as a Source Data file. The remaining panels show representative high-resolution AFM images of the purified DNA self-assembly sample (P). In (**a**), dotted lines on AFM images indicate a composite image made of different sample areas to account for low origami adsorption density. **d** Manual quantification of origami objects on AFM images into “well-folded", “semi-folded" and “mis-folded" categories. The percentage of origami objects in each category is shown.

**Fig. 6:**
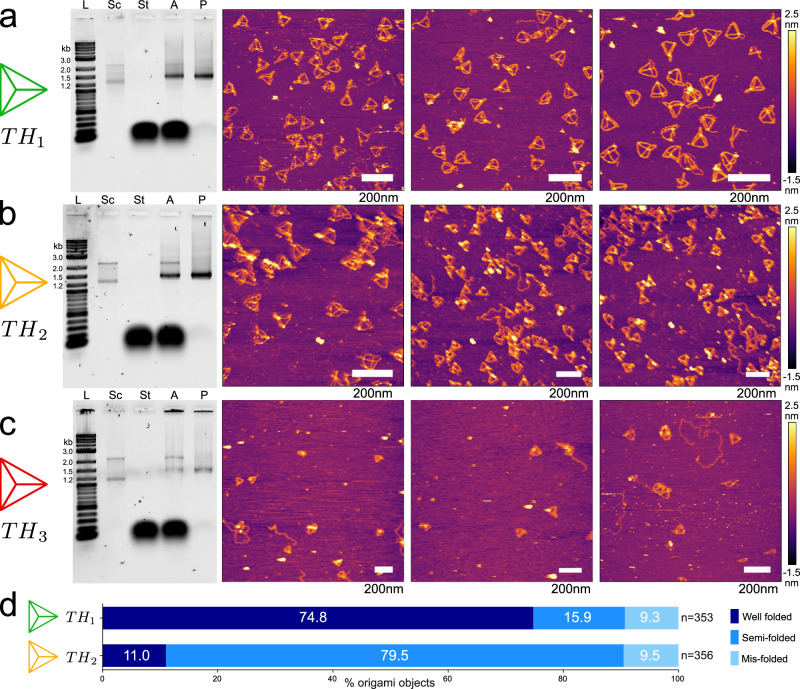
Tetrahedron DNA origami sequence variants: agarose gel and AFM images. **a** Tetrahedron variant *TH*_1_. **b** Tetrahedron variant *TH*_2_. **c** Tetrahedron variant *TH*_3_. In (**a**), (**b**) and (**c**), the first panel shows the laser-scanned image of SYBR® Gold-stained 1% TBE agarose gel with lanes: L = 1kb Ladder; Sc = scaffold only; St = staples only; A = Origami self-assembly reaction mix (scaffold + staples); P = Purified assembly reaction mix used for AFM imaging. The assembly reaction was performed under a fast temperature ramp of 95 °C to 20 °C in 1h 15min (−0.1 °C per cycle, each cycle 6 seconds). All gel electrophoresis experiments were repeated 3 times. Source data are provided as a Source Data file. The remaining panels show representative high-resolution AFM images of the purified DNA self-assembly sample (P). **d** Manual quantification of origami objects on AFM images into “well-folded", “semi-folded" and “mis-folded" categories. The percentage of origami objects in each category is shown: *TH*_3_ is omitted because objects were mis-folded and could not be categorised reliably.

The purified reaction mixtures were analysed by AGE and compared with non-purified samples. The centrifugal filters efficiently separated the higher molecular weight DNA assemblies from staple strands. Purified samples (Figs. 4–6, gel lanes ‘P’) showed the same migration distances as the non-purified samples (Figs. 4–6, gel lanes ‘A’), suggesting that the purification was damage-free.

We classified structures under AFM into three categories: ‘well-folded’ structures resembled the folded shape, ‘semi-folded’ structures resembled the folded shape with small defects, and ‘misfolded’ structures were fragments or aggregates without a specific shape (see Supplementary Note 17).

The origami triangle AFM images showed that *T*_1_ and *T*_2_ variants (Fig. 4a and 4b, respectively) folded with the highest frequency into well-folded triangles (65% and 39.3% respectively). The *T*_3_ variant (Fig. 4c) revealed the lowest percentage of well-folded nanostructures (18.7%) characterised by low stability during the AFM imaging. Semi-folded and misfolded assemblies represented 81.3% of the *T*_3_ origami population. The side average lengths of the *T*_1_ and *T*_2_ variants were compatible with the design, as reported in Supplementary Table 8.

Likewise, the *R*_1_ and *R*_2_ variants (Fig. 5a and 5b, respectively) exhibited higher percentages of well-folded rectangles under AFM (Fig. 5d). Conversely, the *R*_3_ variant (Fig. 5c) showed a high percentage (about 90%) of semi-folded and misfolded assemblies.

It should be noted that blunt end stacking interactions leading to origami dimers and multimers were observed by AFM imaging on *R*_1_ and *R*_2_ variants (Fig. 5a and 5b, respectively). In detail, the *R*_1_ variant often formed longer stacked step-like chains compared to the *R*_2_ variant, with more frequent dimers. Methods to avoid aggregation based on stacking interactions were not present in the origami designs used. The measured length and width of *R*_1_ and *R*_2_ variants (Supplementary Table 8) were consistent with theoretical values (length and width expected values were 62 nm and 47 nm, respectively).

With the 3D tetrahedron variants (Fig. 6), we found that a de Bruijn sequence scaffold (*TH*_1_) gave exceptional assembly yield (74.8% well-folded). Also, there was a clear assembly difference between the best biological sequence (*TH*_2_) and the off-target prone biological sequence (*TH*_3_), with the latter producing mis-folded structures that were not possible to categorise reliably (Fig. 6d).

6-helix bundle origami assembly performed at the University of Bonn showed that the origami variants 6HB_1_ and 6HB_2_ selected as best candidates from the DBS and VEC sequence pools, respectively, had similar high assembly yields (both around 66% well-folded, Supplementary Fig. 43). On the other hand, variant 6HB_3_ displayed significantly less well-folded structures (approx 70% were mis-folded or semi-folded).

### Notes on blind experimental protocol and independent replication

The experimental results described above were obtained following a ‘blind’ experimental protocol. Namely, the computational predictions of which scaffold sequence was a reliable folder (or not) were obscured from the experimentalists at Newcastle. They later carried out the folding experiments, treating all scaffolds in the same manner and analysed their results without an a priori expectation of which scaffold should lead to better AFM results. This blind approach was also replicated independently at the University of Bonn. Bonn’s blind experimental AFM results are in agreement with Newcastle’s for both the triangle and rectangle origami variants (see Supplementary Note 15).

### Probing the triangle DNA origami variants: single-molecule unfolding using optical tweezers

**Fig. 7:**
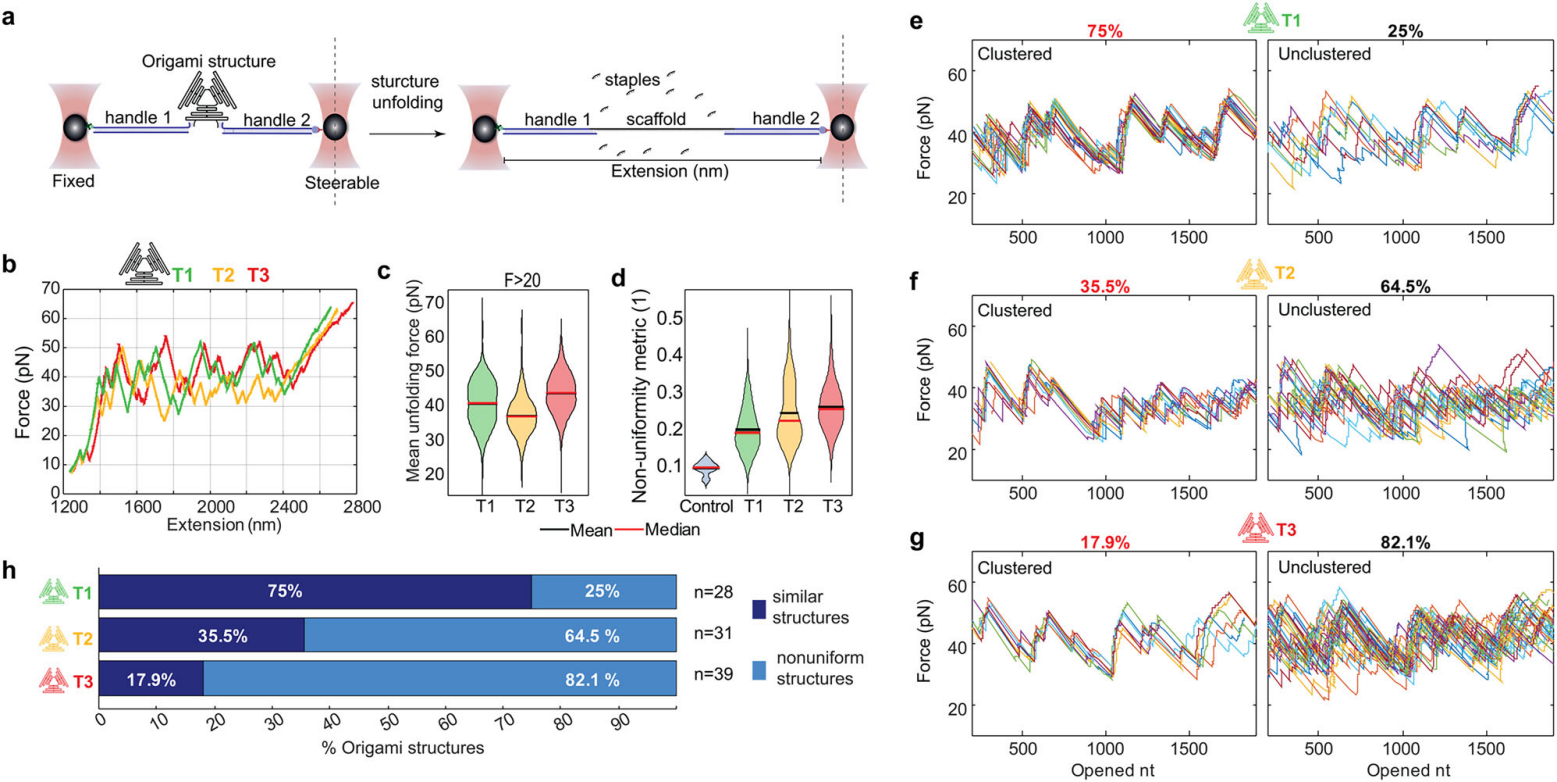
Single-molecule unfolding of DNA origami using optical tweezers. **a** Schematic of the optical tweezers setup. The origami structure is anchored at two points to double-stranded DNA handles, which are attached to polystyrene beads held in optical traps. The distance between the traps is gradually increased, unfolding the origami structure, resulting in a single-stranded DNA (ssDNA) scaffold with staples irreversibly lost to the solution. **b** Representative unfolding force curves are shown for each triangle variant, with different colours representing each, plotted as a function of the extension. **c** Violin plot of unfolding forces above 20 pN, derived from all unfolding curves for each variant (${n}_{{T}_{1}}=30,{n}_{{T}_{2}}=33,{n}_{{T}_{3}}=42$; bars show mean and median). *p*-values obtained from two-sided Student’s T-tests are shown in Supplementary Table 10. **d** Violin plot of the non-uniformity metric, comparing a DNA unzipping control group with the triangle variants, derived from all the unfolding curves that exceed 50% of the scaffold length. (${n}_{{T}_{1}}=28,{n}_{{T}_{2}}=31,{n}_{{T}_{3}}=39$; bars show mean and median). *p*-values obtained from two-sided Student’s t-tests are shown in Supplementary Table 11. **e**–**g** Force vs. opened nucleotides (nt) curves for clustered structures (left) and unclustered structures (right) for each T variant: (**f**) *T*_1_, (**g**) *T*_2_, and (**h**) *T*_3_. **h** Bar plot showing clustering percentages for each group. The non-uniformity threshold for the clusters displayed in this figure is set to 0.14, but the observed clustering is qualitatively insensitive to the choice of threshold (Supplementary Fig. 48d).

To probe their mechanical response with OT (Supplementary Note 18), triangle origamis were prepared using the fast ramp annealing protocol described above but with two modified staples extending from the 5’ and 3’ ends of the scaffold (Methods). These staples were designed with overhangs that facilitate their ligation to two 2kbp dsDNA ‘handles’, each modified at the opposite end with either digoxigenin or biotin. The modified dsDNA handles were then attached to two polystyrene beads, coated with anti-digoxigenin and streptavidin, respectively. Each bead was held in a separate optical trap, with one trap fixed in position and the other steerable (Fig. 7a). By moving the steerable trap at a constant rate, the force applied to the handles and, consequently, to the ends of the origami structure increased. Initially, only the dsDNA handles were stretched. At higher forces, sufficient to perturb the origami structure, we observed a series of sudden force drops accompanied by increases in extension (Fig. 7b, Supplementary Fig. 46), likely the result of the release of a single staple or the cooperative release of a number of them, leading eventually to full disassembly of the origami structure. At this point, the ssDNA scaffold was fully stretched, as confirmed by measuring subsequent force-extension curves for those traces that did not break ( ~ 50%, Supplementary Fig. 47a), which are consistent with the size of the scaffold and the elasticity of ssDNA (Supplementary Fig. 49). The forces required to unfold the origami structures are generally above 30 pN (Supplementary Fig. 47b–c), suggesting that force-induced melting as a result of applying a shearing force between complementary segments (i.e., pulling in the 5’-5’ or 3’-3’ configuration) or stretching one of the complementary segments (i.e., pulling between the 5’ and 3’ ends of the same segment) are responsible for the disruption of the corresponding scaffold-staple interactions^39^. However, we observed a small number of events at forces below 20 pN, mainly at the start of the unfolding reaction (Supplementary Fig. 47b), indicating that some interactions may involve unzipping events, where the 5’ and 3’ ends of the opposite segments are pulled apart^40^. Comparing the different triangle variants (Fig. 7e) reveals a correlation with the construct’s GC content. *T*_3_ (*GC* = 59%) exhibited the highest unfolding forces, *T*_2_ (*GC* = 44.8%) the lowest, and *T*_1_ (*GC* = 50.4) had intermediate unfolding forces.

Notably, there is a significant degree of non-uniformity in the force-extension curves, as evidenced by comparing with a set of curves obtained from unzipping a long DNA hairpin (Supplementary Fig. 46). A higher disorder for the origami is expected, since in contrast to the vectorial DNA unzipping, where there is only a single unfolding pathway possible, multiple pathways likely exist for a DNA origami (Supplementary Fig. 51). However, structural variations resulting from the possibility of off-target binding reactions are likely to contribute to the disorder too, so we expect curves for *T*_1_ and *T*_2_ to be more uniform than those for *T*_3_. To assess the structural nonuniformity of a given origami variant, we defined a nonuniformity metric (NUM) that quantifies the similarity between all possible pairs of traces by calculating the cumulative difference in the position-dependent force (see Methods). As a control, we used DNA unzipping data, which exhibits high uniformity across experiments. NUM values for all origami variants were higher than those of the control, with *T*_1_ showing the lowest mean NUM, followed by *T*_2_, and then *T*_3_ (Fig. 7d). Using the NUM values and a threshold, we then clustered similar traces (see Methods). Remarkably, this analysis revealed that while  ~ 75% of *T*_1_ variants could be clustered into a group of similar traces, and  ~ 36% of the *T*_2_ variants, only  ~ 18% of the traces for *T*_3_ could be clustered (Fig. 7e–h), and this trend was insensitive to the choice of threshold (Supplementary Fig. 48d). These results indicate that *T*_1_ structures are assembled with higher uniformity than the other variants, followed by *T*_2_, and then *T*_3_, consistent with the AFM data.

## Discussion

The AGE and AFM results we obtained from sequence variants of the triangle, rectangle, tetrahedron and 6-helix bundle DNA origamis, and the optical tweezers (OT) results for the triangle origamis, generally confirm our computational predictions of good/mid/bad folding scaffold sequences, and broadly support our main hypothesis that increased off-target binding possibilities have a negative effect on origami assembly yield.

In all cases, origamis prevalent in off-target reactions of all four types (*T*_3_,*R*_3_,*TH*_3_,6HB_3_) nearly completely failed to fold into the intended target shapes in the buffer conditions used (only 0–20% well-folded AFM yield), despite the fact that the staple strands were perfect Watson-Crick complements to the scaffold in all cases. *T*_3_ variants also showed a high degree of non-uniformity in OT experiments, and almost completely failed to cluster into a single uniform group.

As a side note, the extent of off-target reactions exhibited by the “3" variants is also evidenced by the REVNANO tool^41^, which has lower performance when attempting to reverse engineer the sequences for *T*_3_ and *R*_3_ back to their original origami designs.

While assembly yields varied across our different origami experiments, consistent across all experiments was the relationship between biological sequence variants: the “2" variants (either 2D or 3D) always assembled in higher yield than the “3" variants, which largely failed to assemble. This validates our method as a selector of reliably folding biological sequences, given a particular origami design.

Interestingly, in AGE, the *T*_3_ and *R*_3_ variants also displayed well aggregates when only scaffold strands were present (Supplementary Note 14). Moreover, variants *T*_3_, *R*_3_ and *TH*_3_ displayed well aggregates for the full assembly reactions (Figs. 4c, 5c and 6c respectively, lane ‘A’), suggesting that for these origamis, scaffold strands were hybridised together either directly and/or indirectly during assembly.

Therefore, while origami self-assembly undoubtedly has a number of self-correcting error mechanisms to recover from moderate off-target binding ‘noise’ (like strand displacement of off-target bindings by invading on-target staple strands), our results support the view that it is still possible to kinetically trap origami folding if off-target reactions are too prevalent. Our results show that not only pathological sequences, but also regions of commonly used sequences like the Lambda DNA phage can be high in off-target reactions and fail to fold into target origamis when used as scaffold sequences.

Independent replication of folding reactions and AFM experiments at the University of Bonn, Germany (Supplementary Note 15) generally supported the results presented here. Triangle *T*_1_ (de Bruijn sequence) folded with the highest AFM yield, followed by *T*_2_ and then *T*_3_. The same anomaly was also observed for the rectangle origami: *R*_2_ folded with the highest AFM yield, followed by *R*_1_ and then *R*_3_. This anomaly, where *R*_2_ with more off-target reactions assembles at a higher yield than the de Bruijn sequence control *R*_1_ may point to the fact that there may exist other, hidden sequence design principles that can increase assembly yield in the case of specific origami shapes. But we also note that DNA synthesis and purification may be an extra complicating factor for the fair comparison of DBS to biological sequences.

At this stage, it is instructive to reflect on some aspects of our computational method to select origami scaffold sequences, and then proceed to discuss some extensions and limitations of our approach.

A first reflection is that our method is a heuristic approach to increase folding yield for a pre-specified origami design. It indicates which sequences should probabilistically fold better, relative to others, rather than directly predicting yield percentage or directly specifying where fold defects will be located. Our approach works with static sequences, not simulation trajectories of the origami folding pathway, and as such cannot predict classic assembly features such as structure melting temperature, speed of folding, or potential cooling/re-heating hysteresis. However, we note that because our approach rewards sequences which essentially obey domain-level behaviour (i.e., where the only binding events are the intended on-target bindings between staples and scaffold), then our method could be used to select scaffold/staple sequences which are better described by current origami folding models^8,10,30^.

A second important note is that, in the absence of better information, we assume that the four scoring metrics used in Fig. 1 have equal weightings in the final MCDM score for a scaffold sequence. That is, we assume that each different type of off-target binding (staple-scaffold, intra-scaffold, inter-staple, and intra-staple) can equally disrupt the origami folding pathway. Equal weights were sufficient to allow us to predict ‘good’ and ‘bad’ scaffold sequence regions from the lambda DNA sequence for all four DNA origami shapes in this study. However, we expect this weighting could be refined in future if large data sets became available relating sequences and buffer conditions to a uniform measure of origami folding yield. In particular, it seems *M*_2_ would be a suitable candidate to weight more heavily, given that scaffold-scaffold bindings are an intramolecular reaction that occurs at high effective concentration (j-factor), and also because our results evidence that scaffolds can bind inter-molecularly and aggregate.

A third observation is that simpler coarse metrics like scaffold GC content and *k* − *mer* subsequence repeats are indeed correlated with scoring metrics *M*_1_ to *M*_4_, but not tightly enough to substitute any of them (Supplementary Note 7). Unlike the coarse metrics, which are just based on scaffold sequence, scoring metrics *M*_1_ to *M*_4_ are specific to a certain origami design and all four metrics precisely enumerate potential off-target binding sites. Having said that, GC content could still be leveraged to pre-screen scaffold sequences: those sequences outside a 45–55% GC content range could be omitted from the scaffold sequence pool as they are likely to lead to high numbers of sub-sequence repeats and thus higher *M*_1_ and *M*_2_ scores.

Our approach has considerable scope for extension and refinement. Most obviously, the number and type of scoring metrics could be further investigated.

For calculating off-target binding sites with greater accuracy, metrics *M*_1_ and *M*_2_ could use finer-grain averaged energies of base-pairing. Instead of a single per-base pair average binding energy *ΔG*_bp_, two different average free energies *ΔG*_bp_(*GC*) < *ΔG*_bp_(*AT*) could be pre-calculated and employed. This modification could be particularly useful for RNA origami (see below).

Metric *M*_3_ could be modified such that essential staples (e.g., those performing functions like dangling end docking points on origami memory tiles^42^) provide a higher contribution to the *M*_3_ score, thus ensuring that essential staples have a low probability of being co-folded. Additionally, the effect of making *M*_3_ a sum rather than a maximum could be rigorously explored.

To reduce the dimensionality of the objective space and to narrow the Pareto front of solutions, Metric *M*_4_ could conceivably be omitted from the selection step and instead applied post-selection as a filter. This is because strong intra-staple hairpins are actually relatively rare due to (i) the relatively small number of individual staples per structure (with respect to, for example, the number of possible staple-staple co-folds) and (ii) the typically short size of staples. If candidates on the three-dimensional {*M*_1_, *M*_2_, *M*_3_} Pareto front had a high *M*_4_ filter score, they would simply be discarded.

A different benefit metric that rewards stronger on-target staple bindings could potentially be added; then sequence selection would boost favourable interactions (positive design) as well as reduce unfavourable off-target interactions (negative design)^43^. Alternatively, a metric to assist the folding of dense 3D structures could be specified, whereby on-target binding sites destined for the interior of the folded origami object could be rewarded if they were GC-rich, as to increase the probability that they hybridise first before hybridisation is blocked by steric hindrance from folded outer parts.

As each sequence pool considered by our method only provides a sparse sampling of the combinatorially vast sequence space, and considering that even under this sparse sampling significant variation in the metric scores was observed, an alternative sequence sampling technique that couples, e.g., Design of Experiments or generative AI sampling methods with our various metrics might lead to better reliable folders.

Finally, our work may be used as part of a larger pipeline, as a final sequence design stage after staple-routing algorithms^7,37^—which do not take into account off-target reactions—have made an initial acceptable layout of scaffold/staples. Our method is applicable to all origami shapes, whether they be 2D, 3D, grid-based or wireframe-based.

It is also useful to consider the limitations of our method. The first main limitation is computation time. Even though the method works with static sequences, the time to compute the objective space for large origamis (e.g., 7knt scaffold) can be significant on a single CPU (on the order of days) because of the exact enumeration of all off-target binding sites. However, the codebase does allow use of parallel computing in a cluster environment to speed up calculations. In future, smart caching of energy landscapes and/or optimisation of metric *M*_1_ and *M*_2_ scoring code could accelerate performance.

A second limitation is applying our method to larger origamis where the scaffold sequence is fixed and only the scaffold strand can be rotated (a common scenario). As mentioned in the results, strand rotation for larger origamis (thousands of scaffold bases) typically causes negligible variance in the metric scores. As stated, our method is best applied to select origami scaffold sequences from regions of biological sequences which are longer than the origami scaffold. Also, validation on larger multilayer structures with significant fold-order constraints remains an open direction for further studies.

The final challenge is the application to RNA origami. In this work, an initial RNA energy model was also implemented (see Supplementary Note 1). It used a higher average energy per base pair than for DNA ($\Delta {G}_{{{{\rm{bp}}}}}^{{{{\rm{RNA}}}}} < \Delta {G}_{{{{\rm{bp}}}}}^{{{{\rm{DNA}}}}}$) and considered GU pairs as standard base pairs. However, these two factors lead the RNA energy model to identify significantly more off-target sites (in all four categories) for the RNA sequence equivalent version of a DNA origami. A future RNA energy model would benefit from (i) employing not one but three base-pair binding energies $\Delta {G}_{{{{\rm{bp}}}}}^{{{{\rm{RNA}}}}}(GU)$, $\Delta {G}_{{{{\rm{bp}}}}}^{{{{\rm{RNA}}}}}(AT)$, $\Delta {G}_{{{{\rm{bp}}}}}^{{{{\rm{RNA}}}}}(GC)$, each averaged over all possible nearest neighbour combinations, to make off-target site identification more accurate and (ii) experimental testing of RNA origami self-assembly, as done for DNA origami in this study. A hybrid DNA/RNA thermodynamic model tailored for origami assembly under physiological conditions could also be parameterised in future work^44^.

Computational efforts to improve DNA origami nanotechnology have traditionally focused on optimising origami scaffold and staple strand routings in the origami design, rather than on primary base sequence optimisation. Nevertheless, base sequences are able to cause long-range off-target interactions between multiple components of the system, which complicate and potentially trap the self-assembly process.

In this study, we focused exclusively on off-target reactions and developed a multi-objective computational method to select scaffold sequences that simultaneously minimise four different types of off-targets. We demonstrated that off-target side reactions during self-assembly can significantly affect the final AFM yield of four different target origami shapes, despite perfect Watson-Crick complementarity existing between scaffold and staples. We also showed that scaffold sequences for the same designed shape, which are optimised to different degrees by our method, have, under single-molecule optical tweezer experiments, distinct degrees of uniformity in their force profiles, which are linked to our computational predictions.

Overall, this work proposes a change in perspective about DNA origami base sequence. Rather than a top-down engineering view, we advance a more bottom-up systems view of the origami self-assembly process. Instead of the base sequence being seen as a benign low-level implementation of the desired staple-scaffold domain-level bindings, this study suggests that potential off-target interactions inherent in the particular base sequences employed should be taken more seriously, particularly when biological scaffold sequences are used.

## Methods

### Blinded experiments

DNA origami folding reactions were carried out with blinded samples to mitigate experimental bias.

### Materials and reagents

All DNA oligonucleotides and gBlocks Gene Fragments were purchased from IDT. The DNA oligonucleotides were resuspended in UltraPure DNase/RNase-free distilled water (Thermo Fisher Scientific) to give stock solutions of 100 μM and stored at −20 °C. The gBlocks Gene Fragments were resuspended in Tris-EDTA (TE) buffer at a final concentration of 10 ng/*μ*L and stored at −20 °C.

EDTA 0.5M pH 8.0, DNase/RNase-free Tris 1M pH 8.0, SYBR^TM^ Gold nucleic acid gel stain, UltraPure 10X TBE buffer, 10X TAE buffer molecular biology grade (400 mM Tris and 0.01 M EDTA), Ambion®10X gel loading solution were purchased from Thermo Fisher Scientific.

Q5®High-Fidelity 2X Master Mix, Monarch®PCR & DNA Clean-up kit, 1 kb and 1 kb Plus DNA ladders, Gel loading dye purple (6X), T7 exonuclease and Lambda DNA (500*μ*g/mL) were purchased from NEB.

Agarose, Nancy-520, magnesium acetate BioUltra 1M in water, Amicon®Ultra 100 kDa centrifugal filters were purchased from Sigma-Aldrich.

### Double-stranded DNA amplification, purification, and agarose gel electrophoresis

Strand-specific T7 exonuclease digestion was employed to synthesise tailor-made single-stranded DNA sequences (ssDNA) to be used as scaffolds in the folding reactions of all twelve origami variants (three triangles, three rectangles, three helix bundles and three tetrahedra). Phosphorothioate protective modification at the 5’ end of the desired ssDNA strand was introduced by polymerase chain reaction (PCR) using a modified forward primer (Supplementary Note 12) as previously reported^38^. In detail, the double-stranded DNAs (dsDNA) of defined sequence and length were amplified from Lambda DNA or gBlocks Gene Fragments templates using a modified forward primer which contains sequential phosphorothioate bonds between the first five nucleotides. The modified phosphate backbone inhibited exonuclease digestion, while the non-required antisense strand was digested by T7 exonuclease.

Double-stranded gBlocks Gene Fragments (De Bruijn sequences) and Lambda DNA were amplified using Q5®High-Fidelity DNA polymerase 2X master mix, 10*μ*M of 5’- phosphorothioate modified forward primer and 10μM of reverse primer (Supplementary Note 12): the final concentration of each primer was 0.5μM in 50μL of final reaction volume. When the origami centrifugal purification and imaging were considered, a total of 20 PCR tubes were prepared for each DNA origami variant. To ensure a successful PCR for each template, the annealing temperature, annealing duration, number of cycles and template amount were optimised (Supplementary Note 12). For high melting temperature primer pairs, a 2-step PCR was run combining annealing and extension (Lambda *T*_3_ and gBlocks *T*_1_ in Supplementary Table 5). The PCR product was purified using Monarch®PCR & DNA Cleanup kit: PCR solutions diluted with DNA cleanup binding buffer were loaded onto 4 distinct columns, washed, and eluted in 10*μ*L of UltraPure DNase/RNase free distilled water.

The DNA concentration of the total eluted volume ( ≈ 32*μ*L) was measured using a NanoDrop One/OneC spectrophotometer. After the addition of the purple gel loading dye (2*μ*L) to 10*μ*L of TE containing 100ng of purified dsDNA, the size of purified amplicons were evaluated on 1% agarose gel in TBE for 1h at 110V: the gels were pre-stained with Nancy-520, visualised using Typhoon laser scanner (*λ*_exc_ 488nm, *λ*_em_ 532nm, PMT voltage 380, normal sensitivity, GE Healthcare Life Sciences) and ImageQuant TL software (GE Healthcare Life Sciences). The 1 kb DNA ladder (NEB) was used as a molecular weight marker.

### Purified double-stranded DNA digestion using T7 exonuclease, purification, and agarose gel electrophoresis

Amplified and purified dsDNA template was used for ssDNA preparation, as previously described^38^ with some modifications. Briefly, 6 digestion mixes were prepared for each variant: one reaction mix contained 20 units (*T*_1_, *T*_2_), 25 units (*T*_3_, *R*_2_) or 30 units (*R*_3_, *R*_1_) of T7 exonucleases, 5*μ*L NEB Buffer 4, 500ng of purified dsDNA template and UltraPure DNase/RNase-free distilled water (up to 50*μ*L). Similarly, purified dsDNA templates for 3D origami scaffold preparation were digested with 20–30 units of T7 exonucleases. After overnight incubation at 25^∘^C, the ssDNA product was purified using Monarch®PCR & DNA Clean-up kit: digested solutions diluted with DNA cleanup binding buffer were loaded onto 3 distinct columns, washed, and eluted in 12*μ*L of UltraPure DNase/RNase free distilled water. The ssDNA concentration of the total eluted volume ( ≈ 30*μ*L) was measured using a NanoDrop One/OneC spectrophotometer. After the addition of the purple gel loading dye, the purified ssDNA was run on a 1% agarose gel in TBE and imaged as described above. The ssDNA solution was kept at -20^∘^C until the origami folding reaction (the day after). Each purified ssDNA scaffold was sent for Sanger sequencing (Eurofins Genomics) and aligned using the SnapGene®software.

### 2D and 3D DNA origami folding reactions and pre-screening by agarose gel electrophoresis

For each DNA origami variant folding, each ssDNA staple strands set (final concentration 16nM each) was mixed in a 10-fold excess with complementary ssDNA scaffold (1.6nM) in 50*μ*L of folding buffer (1x TAE molecular biology grade buffer, 40mM Tris Acetate, pH 8.3, 1mM EDTA, supplemented with 12.5mM BioUltra magnesium acetate) as previously reported^20^. Folding solutions and negative controls (only scaffold and only staple strand samples) for each variant were incubated at 4 different temperature conditions and then held at constant temperature (4 °C) to stop the reaction. The tested temperature conditions were: (i) isothermal folding at 37 °C for 1 h 15 min without initial denaturation; (ii) isothermal folding at 37 °C for 1 h 15 min with an initial denaturation at 95 °C for 1 min; (iii) fast temperature ramp from 95 °C to 20 °C in 1 h 15 min (−0.1 °C/cycle, each cycle 6 s); (iv) slow temperature ramp from 95 °C to 20 °C in 5 h 40 min (−0.1 °C/cycle, each cycle 27 s).

Scaffold, staple strands, and DNA origami samples (10*μ*L) were run on a 1% agarose gel in 1x TBE buffer at 90 V for 1 h 20 min at low temperature (below 10 °C). Samples annealed considering the first 3 temperature ramps (isothermal folding with or without initial denaturation and fast ramp) were kept at 4 °C and run again with samples annealed with a slower ramp the day after. The gels were stained in 1x TBE containing SYBR®Gold for 15 min under gentle agitation at 15 rpm (Gyro-rocker SSL3, Stuart) and visualised using a Typhoon laser scanner. The 1 kb and 1 kb Plus DNA ladders were used as molecular weight markers. To monitor the structural stability of the assemblies over time, the gel electrophoretic analysis was repeated after 4/5 days on all samples stored at 4 °C. 3D DNA origami folding reactions under a fast temperature ramp and pre-screening by agarose gel electrophoresis were conducted as mentioned above.

### DNA origami purification and AFM imaging

The folded constructs at a specific selected temperature condition were purified from excess staple strands using the Amicon®Ultra 0.5 mL 100 kDa centrifugal filters. Samples were added to the filter device and centrifuged at 14,000 g for 1 min at 11 °C. The flowthrough was removed and the filter was washed 4 times (14,000 g for 1 min at 11 °C) with 450*μ*L of 1x folding buffer. To recover the purified and concentrated sample, the filter was turned upside down and centrifuged at 1000 g for 5 min at 11 °C. Scaffold, staple strands, non-purified and purified DNA origamis were run on 1% agarose gel in 1x TBE buffer, stained and visualised using a Typhoon laser scanner as previously reported.

Purified DNA origami samples were stored at 4 °C and imaged by AFM as follows. Freshly cleaved mica (Mica Grade V-4 12 mm Discs x 0.15 mm, Azpack Ltd., Loughborough, UK) was passivated using 1x folding buffer. After three washing steps with folding buffer (60μL each), the purified construct solution was added to the passivated mica surface, allowed to absorb for 1–2 min and imaged immediately ( ≈ 100μL of the folding buffer was added after the origami absorption). The purified assemblies were characterised in tapping mode in liquid using VRS Cypher ES AFM (Asylum Research, Oxford Instruments, Santa Barbara, CA, USA). The vertical oscillation of the BioLever Mini probe (spring k of 0.09 N/m, Asylum Research, Oxford Instruments, Santa Barbara, CA) was controlled by photothermal excitation (Blue Drive) and the imaging was conducted at 25.0  ± 0.1 °C.

Large scan size (about 1 × 1 μm) and zoomed scans were considered to count well-folded, folded with defects (or semi-folded in the text) and unfolded assemblies. All the images were corrected for tilt (line or plane flattening) and lightly low-pass filtered to remove high-frequency noise, using the Asylum Research analysis package for Igor Pro(R) (Wavemetrics, OR, USA)^45^.

### DNA origami: folding reactions, purifications and AFM imaging conducted at University of Bonn, Germany

To validate our approach, data from another nano biotech laboratory was considered. The blind experiments protocol was uploaded in protocols.io^46^ to share an up-to-date version. In detail, the amplified and purified dsDNA templates were digested for 4 h instead of overnight, and the AFM imaging was modified as follows. Freshly cleaved mica was passivated using poly-L-ornithine instead of 1X folding buffer. After samples deposition, high-resolution AFM images were obtained using a JPK NanoWizard®3 Ultra AFM (JPK Instruments, Berlin). The USC-F0.3-k0.3-10 ultra-short AFM tips (NanoWorld Innovative Technologies, Switzerland) with a nominal force constant of 0.3 N/m were used. Examples of ‘semi-folded’ and ‘mis-folded’ categories were shared by Newcastle to allow a consistent quantification of assemblies (see Supplementary Note 17).

### Optical-tweezers experiments

*Molecular constructs*. Experiments were conducted using a custom-built double-trap optical tweezers setup, as previously described^47^. Constructs to probe the force response of the origamis were based on previous designs aimed at probing protein-DNA interactions^48^^49^, with modifications. We generated two 2000 bp DNA handles, each uniquely tagged with double digoxigenin or biotin, by performing PCR on bacteriophage lambda DNA. The biotin handle was created using a biotin-tagged forward primer, while the digoxigenin handle used a forward primer containing a BglI restriction site to create an overhang for ligation with two annealed, commercially purchased oligos featuring 3’ and 5’ digoxigenin terminal modifications (IDT, Supplementary Table 13). The reverse primers were designed with restriction sites for NcoI and BglI for the biotin- and digoxigenin-tagged handles, respectively. Digestion with NcoI-HF and BglI (New England Biolabs) was done following the manufacturer’s recommended protocol, resulting in distinct overhangs for each handle. For the optical tweezers experiment, origami structures were designed with complementary overhangs at both scaffold ends, matching the overhangs on the two DNA handles. The origami structures were first assembled as described above. Subsequently, the handles were ligated to the origami structures at an equimolar ratio using a rapid ligase system (Promega), with incubation at 16^∘^C for 30 minutes.

*Force-extension curves acquisition*. The full construct (handles + origami structure) was incubated for 15 minutes on ice with 0.8 *μ*m polystyrene beads (Spherotech) coated with anti-digoxigenin antibodies. The reaction was then diluted 1000-fold in a working buffer (10 mM Tris-Cl pH 7.4, 150 mM NaCl, 1.5 mM MgCl_2_, 3% glycerol, and 0.01% bovine serum albumin). Tether formation was conducted in situ within the experimental chamber at room temperature by capturing an anti-digoxigenin bead bound to the DNA construct in one optical trap and a 0.9 *μ*m streptavidin-coated polystyrene bead in the other trap. The two beads were brought into close proximity to allow binding between the biotin tag on the DNA and the streptavidin on the bead (Fig. 7a). Next, the distance between the two beads was gradually increased at a constant rate until reaching a maximal force of 65 pN to fully unfold the origami structure. If the tether remained intact, the distance between the beads was decreased and then increased again, to reveal the stretching of the scaffold and the existence of any possible residual structure (Supplementary Fig. 49).

*Data analysis*. Data were digitised at a 2500 Hz sampling rate and saved to disk, with all subsequent data processing performed in MATLAB (MathWorks). From the measured tether extension and force, the extension due to dsDNA handles was calculated and subtracted at each time point (Supplementary Fig. 50b). The resulting extension was then divided by the extension of one ssDNA base (calculated from the measured force using the worm-like-chain model) to yield the number of opened nucleotides (Supplementary Fig. 50b). Data collection statistics are summarised in Supplementary Table 9. The nonuniformity metric (NUM) was calculated for each pair of traces obtained within the same variant type. For each pair, we low-pass filtered the data to 10 Hz. The NUM (Eq. (1)) was defined as the root of the mean squared force difference, normalised by the mean peak-to-peak force difference for each variant (Supplementary Table 12):

1 $${{{\rm{NUM}}}}=\frac{\sqrt{\frac{{\sum }_{i=1}^{n}\delta {{f}_{i}}^{2}}{n}}}{\Delta F}$$

To account for instrumental variability between different unfolding curves, we scanned the NUM for each pair by shifting the two traces along the open nt axis ( ± 150 nt in 5-nt increments) and along the unfolding force axis ( ± 3 pN in 0.5-pN increments). The lowest NUM value was assigned to each pair of curves. Clustering of similar traces is achieved by first connecting traces with a NUM below a specified threshold (0.14 in Fig. 6e-h), and then grouping traces that share at least one overlapping connection. To qualify as a cluster, a group must consist of at least three traces; groups with fewer traces are not considered clusters (Fig. 6e–g). If more than one cluster existed, the largest resulting cluster was chosen, and traces that belonged to other groups were classified as unclustered. The unfolding curves shown in Fig. 7e–g were aligned to a randomly chosen reference curve from the similar trace cluster, using the shift along the open nt and unfolding force axes that yielded the lowest NUM value.

### Reporting summary

Further information on research design is available in the Nature Portfolio Reporting Summary linked to this article.

## Supplementary information

Supplementary Information

Reporting Summary

Transparent Peer Review file

## Source data

Source Data

## Data Availability

Gels (uncropped) source data are provided with this paper in a source data file, other data are available via the supplementary information file. Computed data (e.g., Fig. 1 (e, f), 3 (b, c, d, e)) are fully reproducible via the Python source code provided. A Matlab file that contains optical tweezers data has been deposited in Zenodo at 10.5281/zenodo.19347172. Electronic scadnano designs (.sc) of all DNA origamis used in the paper, DNA sequences used and scaffold selector HTML reports for all DNA origamis in the paper are available at Zenodo 10.5281/zenodo.17273772. Source data is available for Figs. 4, 5, 6, 7c–d in the associated source data file. Source data are provided with this paper.

## Code availability

Python source code (Academic Noncommercial License) and documentation are located at https://scaffoldselector.readthedocs.io. A permanent snapshot of the source code and scadnano designs of the origami variants used is located in Zenodo at 10.5281/zenodo.17273772.

## References

[1] Dey, S. et al. DNA origami. *Nat. Rev. Methods Prim.* **1**, 13 (2021).

[2] Nie, Z. (ed.) *DNA Nanotechnology for Cell Research: From Bioanalysis to Biomedicine* (Wiley, 2024) https://www.wiley.com/en-us/DNA+Nanotechnology+for+Cell+Research%3A+From+Bioanalysis+to+Biomedicine-p-9783527351732.

[3] Li, L., Nie, S., Du, T., Zhao, J. & Chen, X. DNA origami technology for biomedical applications: Challenges and opportunities. *MedComm - Biomater. Appl.* **2**, https://onlinelibrary.wiley.com/doi/10.1002/mba2.37 (2023).

[4] Keller, A. & Linko, V. Challenges and perspectives of DNA nanostructures in biomedicine. *Angew. Chem. Int. Ed.* **59**, 15818–15833 (2020). 10.1002/anie.201916390 PMC7540699 32112664

[5] Jaekel, A., Lill, P., Whitelam, S. & Saccà, B. Insights into the structure and energy of DNA nanoassemblies. *Molecules* **25**, 5466 (2020). 33255286 10.3390/molecules25235466 PMC7727707

[6] Dunn, K. E. et al. Guiding the folding pathway of DNA origami. *Nature* **525**, 82–86 (2015). 26287459 10.1038/nature14860

[7] Aksel, T., Navarro, E., Fong, N. & Douglas, S. Design principles for accurate folding of DNA origami. *Proc. Natl. Acad. Sci. USA* **121**. 10.1073/pnas.2406769121 (2024). 10.1073/pnas.2406769121 PMC11621765 39570311

[8] DeLuca, M. et al. Mechanism of DNA origami folding elucidated by mesoscopic simulations. *Nat. Commun.* **15**, 3015 (2024). 38589344 10.1038/s41467-024-46998-y PMC11001925

[9] Ke, Y., Bellot, G., Voigt, N. V., Fradkov, E. & Shih, W. M. Two design strategies for enhancement of multilayer-DNA-origami folding: underwinding for specific intercalator rescue and staple-break positioning. *Chem. Sci.* **3**, 2587 (2012). 24653832 10.1039/C2SC20446K PMC3957201

[10] Cumberworth, A., Reinhardt, A. & Frenkel, D. Lattice models and Monte Carlo methods for simulating DNA origami self-assembly. *J. Chem. Phys.*** 149**, 234905-234905 (2018). 10.1063/1.5051835 30579289

[11] Sobczak, J.-P. J., Martin, T. G., Gerling, T. & Dietz, H. Rapid folding of DNA into nanoscale shapes at constant temperature. *Science* **338**, 1458–1461 (2012). 23239734 10.1126/science.1229919

[12] Rossi-Gendron, C. et al. Isothermal self-assembly of multicomponent and evolutive DNA nanostructures. *Nat. Nanotechnol.* **18**, 1311–1318 (2023). 37524905 10.1038/s41565-023-01468-2 PMC10656289

[13] Wei, X., Nangreave, J., Jiang, S., Yan, H. & Liu, Y. Mapping the thermal behavior of DNA origami nanostructures. *J. Am. Chem. Soc.* **135**, 6165–6176 (2013). 23537246 10.1021/ja4000728

[14] Majikes, J. M., Patrone, P. N., Kearsley, A. J., Zwolak, M. & Liddle, J. A. Failure mechanisms in DNA self-assembly: barriers to single-fold yield. *ACS Nano* **15**, 3284–3294 (2021). 33565312 10.1021/acsnano.0c10114 PMC11005093

[15] Rodriguez, A. et al. Self-assembly of DNA nanostructures in different cations. *Small* **19**, 10.1002/smll.202300040 (2023). 10.1002/smll.202300040 PMC10538431 37264756

[16] Enlund, E., Julin, S., Linko, V. & Kostiainen, M. A. Structural stability of DNA origami nanostructures in organic solvents. *Nanoscale* http://pubs.rsc.org/en/Content/ArticleLanding/2024/NR/D4NR02185A (2024). 10.1039/d4nr02185a PMC11256221 38910453

[17] Engelhardt, F. A. S. et al. Custom-size, functional, and durable DNA origami with design-specific scaffolds. *ACS Nano* **13**, 5015–5027 (2019). 30990672 10.1021/acsnano.9b01025 PMC6992424

[18] Arbona, J.-M., Aimé, J.-P. & Elezgaray, J. Cooperativity in the annealing of DNA origamis. *J. Chem. Phys.*** 138**, https://pubs.aip.org/jcp/article/138/1/015105/191997/Cooperativity-in-the-annealing-of-DNA-origamis (2013). 10.1063/1.4773405 23298065

[19] Kosinski, R. et al. Sites of high local frustration in DNA origami. *Nat. Commun.* **10**, 1061 (2019). 30837459 10.1038/s41467-019-09002-6 PMC6400978

[20] Rothemund, P. W. K. Folding DNA to create nanoscale shapes and patterns. *Nature* **440**, 297–302 (2006). 16541064 10.1038/nature04586

[21] Williams, S. et al. Tiamat: A three-dimensional editing tool for complex dna structures. In Goel, A., Simmel, F. C. & Sosík, P. (eds.) *DNA Computing* 90–101 (Springer Berlin Heidelberg, Berlin, Heidelberg, 2009).

[22] de Bruijn, N. G. A combinatorial problem. *Proc. Sect. Sci. K. Nederlandse Akademie van. Wetenschappen te Amst.* **49**, 758–764 (1946).

[23] Kozyra, J. et al. Designing uniquely addressable bio-orthogonal synthetic scaffolds for DNA and RNA origami. *ACS Synth. Biol.* **6**, 1140–1149 (2017). 28414914 10.1021/acssynbio.6b00271

[24] Parsons, M. F. et al. 3D RNA-scaffolded wireframe origami. *Nat. Commun.* **14**, 382 (2023). 36693871 10.1038/s41467-023-36156-1 PMC9872083

[25] Lee Tin Wah, J., David, C., Rudiuk, S., Baigl, D. & Estevez-Torres, A. Observing and controlling the folding pathway of DNA origami at the nanoscale. *ACS Nano* **10**, 1978–1987 (2016). 26795025 10.1021/acsnano.5b05972

[26] Snodin, B. E. K. et al. Direct simulation of the self-assembly of a small DNA origami. *ACS Nano* **10**, 1724–1737 (2016). 26766072 10.1021/acsnano.5b05865

[27] Bush, J. et al. Synthesis of DNA origami scaffolds: current and emerging strategies. *Molecules* **25**, 3386 (2020). 32722650 10.3390/molecules25153386 PMC7435391

[28] Torelli, E. et al. Cotranscriptional folding of a bio-orthogonal fluorescent scaffolded RNA origami. *ACS Synth. Biol.* **9**, 1682–1692 (2020). 32470289 10.1021/acssynbio.0c00009

[29] Wu, H. et al. Expanding DNA origami design freedom with de novo synthesized scaffolds. *J. Am. Chem. Soc.* **146**, 16076–16084 (2024). 38803270 10.1021/jacs.4c03148

[30] Dannenberg, F. et al. Modelling DNA origami self-assembly at the domain level. *J. Chem. Phys.* **143**, https://pubs.aip.org/jcp/article/143/16/165102/75210/Modelling-DNA-origami-self-assembly-at-the-domain (2015). 10.1063/1.4933426 26520554

[31] Taneda, A. Multi-objective optimization for RNA design with multiple target secondary structures. *BMC Bioinforma.* **16**, 280 (2015). 10.1186/s12859-015-0706-x PMC4559319 26335276

[32] Nanda, V., Belure, S. V. & Shir, O. M. Searching for the Pareto frontier in multi-objective protein design. *Biophys. Rev.* **9**, 339–344 (2017). 28799089 10.1007/s12551-017-0288-0 PMC5578931

[33] Otero-Muras, I. & Banga, J. R. Automated design framework for synthetic biology exploiting Pareto optimality. *ACS Synth. Biol.* **6**, 1180–1193 (2017). 28350462 10.1021/acssynbio.6b00306

[34] Papathanasiou, J. & Ploskas, N. *Multiple Criteria Decision Aid: Methods, Examples and Python Implementations* (Springer, 2018).

[35] Fornace, M. E., Porubsky, N. J. & Pierce, N. A. A unified dynamic programming framework for the analysis of interacting nucleic acid strands: enhanced models, scalability, and speed. *ACS Synth. Biol.* **9**, 2665–2678 (2020). 32910644 10.1021/acssynbio.9b00523

[36] Brown, S. et al. An easy-to-prepare mini-scaffold for DNA origami. *Nanoscale* **7**, 16621–16624 (2015). 26413973 10.1039/c5nr04921k

[37] Jun, H. et al. Rapid prototyping of arbitrary 2d and 3d wireframe DNA origami. *Nucleic Acids Res.* **49**, 10265–10274 (2021). 34508356 10.1093/nar/gkab762 PMC8501967

[38] Noteborn, W. E. M., Abendstein, L. & Sharp, T. H. One-pot synthesis of defined-length ssDNA for multiscaffold DNA origami. *Bioconjugate Chem.* **32**, 94–98 (2021). 10.1021/acs.bioconjchem.0c00644 PMC7830112 33307668

[39] Hatch, K., Danilowicz, C., Coljee, V. & Prentiss, M. Demonstration that the shear force required to separate short double-stranded DNA does not increase significantly with sequence length for sequences longer than 25 base pairs. *Phys. Rev. E* **78**, 011920 (2008). 10.1103/PhysRevE.78.011920 18763995

[40] Bockelmann, U., Thomen, P., Essevaz-Roulet, B., Viasnoff, V. & Heslot, F. Unzipping DNA with optical tweezers: high sequence sensitivity and force flips. *Biophys. J.* **82**, 1537–1553 (2002). 11867467 10.1016/S0006-3495(02)75506-9 PMC1301953

[41] Shirt-Ediss, B. et al. Reverse engineering DNA origami nanostructure designs from raw scaffold and staple sequence lists. *Comput. Struct. Biotechnol. J.* **21**, 3615–3626 (2023). 37520280 10.1016/j.csbj.2023.07.011 PMC10371787

[42] Dickinson, G. D. et al. An alternative approach to nucleic acid memory. *Nat. Commun.* **12**, 2371 (2021). 33888693 10.1038/s41467-021-22277-y PMC8062470

[43] Dirks, R. Paradigms for computational nucleic acid design. *Nucleic Acids Res.* **32**, 1392–1403 (2004). 14990744 10.1093/nar/gkh291 PMC390280

[44] Banerjee, D. et al. Improved nearest-neighbor parameters for the stability of RNA/DNA hybrids under a physiological condition. *Nucleic Acids Res.* **48**, 12042–12054 (2020). 32663294 10.1093/nar/gkaa572 PMC7708073

[45] Beton, J. G. et al. TopoStats—a program for automated tracing of biomolecules from AFM images. *Methods* **193**, 68–79 (2021). 33548405 10.1016/j.ymeth.2021.01.008 PMC8340030

[46] Teytelman, L., Stoliartchouk, A., Kindler, L. & Hurwitz, B. L. Protocols.io: virtual communities for protocol development and discussion. *PLOS Biol.* **14**, e1002538 (2016). 27547938 10.1371/journal.pbio.1002538 PMC4993360

[47] Malik, O., Khamis, H., Rudnizky, S., Marx, A. & Kaplan, A. Pausing kinetics dominates strand-displacement polymerization by reverse transcriptase. *Nucleic Acids Res.* **45**, 10190–10205 (2017). 28973474 10.1093/nar/gkx720 PMC5737391

[48] Rudnizky, S. et al. Single-molecule DNA unzipping reveals asymmetric modulation of a transcription factor by its binding site sequence and context. *Nucleic Acids Res.* **46**, 1513–1524 (2018). 29253225 10.1093/nar/gkx1252 PMC5815098

[49] Rudnizky, S. et al. Extended and dynamic linker histone-DNA interactions control chromatosome compaction. *Mol. Cell* **81**, 3410–3421.e5 (2021). 34192510 10.1016/j.molcel.2021.06.006

[50] Poppleton, E., Romero, R., Mallya, A., Rovigatti, L. & Šulc, P. OxDNA.org: a public webserver for coarse-grained simulations of DNA and RNA nanostructures. *Nucleic Acids Res.* **49**, W491–W498 (2021). 34009383 10.1093/nar/gkab324 PMC8265093

