# Supplementary material for: Optimising DNA origami assembly by reducing off-target interactions
Source: Nat Commun. 2026 May 26;17:6859. doi: 10.1038/s41467-026-73387-4 (PMC13388973; doi:10.1038/s41467-026-73387-4)
Supplement: Supplementary file 3 — Transparent Peer Review file [file 41467_2026_73387_MOESM3_ESM.pdf]

nature portfolio

Peer Review File

Optimising DNA origami assembly by reducing off-target interactions

Corresponding Author: Professor Natalio Krasnogor

This file contains all reviewer reports in order by version, followed by all author rebuttals in order by version.

Attachments originally included by the reviewers as part of their assessment can be found at the end of this file.

Version 0:

Reviewer comments:

Reviewer #1

(Remarks to the Author)
Shirt-Ediss et al. present work in which several metrics are proposed for assessing the likley success of DNA origami folding. These metrics are simplified estimates of the off-target staple binding to the scaffold, scaffold-scaffold binding, staple-staple binding and self-binding of a single staple. The authors discuss how variable these metrics are for a range of origami designs and a range of methods for selecting the scaffold (extracts from biological vectors, random, and a "De Bruijn pool" in which sequences of length greater than or equal to k, with k=5 or 6, are never repeated). They find that biological vectors produce the largest variance over these metrics, and also that they tend to score worse on the first two metrics at least. The authors suggest that the low variance of random and De Bruijn pools suggest that sequence selection is not necessary in those cases.

The authors then tested their metrics by designing 3 variants (optimized De Bruijn, optimized biological vector and pathological biological vector) of 2 origami shapes (triangle and rectangle). In each case, the De Bruijn sequence is predicted to outperform the optimized biological vector, which is predicted to outperform the pathological biological vector. The authors perform folding experiments and demonstrate that this behaviour is observed, except for the fact that the optimized biological vector slightly out-performs the optimized De Bruijn sequence. Finally, force-extension data is reported for the three triangular designs, with traces being more uniform for the De Bruijn sequence and less uniform for the pathological biological vector, suggesting a more disparate ensemble of folded structures.

The authors address an interesting question appropriate for the broad readership of Nature Communications. The metrics are an interesting attempt to simplify the problem of identifying off-target interactions in origami assembly. The theory is combined with two types of experiment to assess its predictive power. The manuscript is well written and generally easy to follow. On that basis I am minded to support the progress of the work towards publication. My main concern is the degree to which the theoretical predictions have been robustly tested.

Main concern:
1. The authors have only assessed their system using the folding of two origami shapes, with three designs each, and by looking at the force-extension properties of three designs of the same shape. The results are encouraging, but this is a fairly small sample - we certainly cannot, at the moment, be confident that the metrics work in general. It is worth noting that the two biological vectors considered have been selected from the pareto front and reverse front of the metrics -- in some sense, providing the easiest challenge for discrimination. It is less clear how reliable the authors approach would be in improving folding relative to a typical biological vector.

Significant comments:
2. The motivation for some aspects of the model design were a bit unclear to me.

(a) Why do the authors use totals for M1 and M2, but only worst single case for M3 and M4?

(b) Why use a sequence-averaged model? Is the extra cost of including the specific base pair free energies (see https://pubmed.ncbi.nlm.nih.gov/15139820/) significant?

(c) When using the sequence-averaged model, why not just directly average the published parameters (see https://pubmed.ncbi.nlm.nih.gov/15139820/) rather than try to reverse engineer them by averaging over nupack outputs? Are the results obtained significantly different from taking this approach?

(d) When the authors say (in the SI) that the absolute value of dGs were used, is it true that all dGs entering in to the metrics are negative? Otherwise there is a slightly strange effect where a really positive free energy (which is irrelevant to folding) would be interpreted as equivalent to really stron gbinding?

(e) In general, the authors don't try to weight the metrics relative to one another in a careful way. Nonetheless, it is worth noting that it would be possible to do a more careful alignment of M3 and M4. M4 is an intramolecular folding and M3 is an intermolecular association, with the strands typically at concentrations of 10s of nanomolar. The same standard free energy dG0 for both corresponds to a very different likelihood of problems. For M4, the likelihood of a structure relative to the unfolded state is exp(-dG0/RT). For M3, the likelood of a duplex relative to separated strands is (very roughly) [concentration of strands]/[reference concentration] * exp(-dG0). Since the reference concentration of Nupack is 55M (see the original Dirks et al paper), the ratio [concentration of strands]/[reference concentration] is of order 10^9. This is a massive difference that really should feature in the metrics. In practice, it won't change the pareto front but would it change some of the analysis that the auhtors do? To a lesser extent this is also true of the scaffold self-folding versus the staple mis-bonding, but the authors are not carefully calculating looping costs for the scaffold anyway, so the direct comparison can't be made.

2. The authors present an analysis of the inaccuracy of using their sequence-averaged description, but not the cost of ignoring asymmetric loops. Is it possible to estimate that, for small systems at least?

3. The main text contains quite a lot of discussion of data that I think is only presented in the SI (see p6, p8, p12). This makes it quite hard to follow some of the arguments - data relegated to the SI should, ideally, not require a detailed discussion.

4. Was the classification of structures performed by hand? Are there details on the systematic approach used to classify? What were the original rules followe dby those in Newcastle?

Minor comments:
5. What do the coloured blocks in Fig 1 represent?

6. What is the motivation for the different experimental protocols used for different systems (see first column, p15, and Supplementary Table 5).

7. Perhaps I missed it, but I was unsure how the scaffold strand for the De Bruijn system was obtained

8. On p12, the authors discuss "received wisdom" that any scaffold will fold. Are there any sources to back this up?

9. The meaning of the "threshold (caption of fig 14) was not that obvious from the main text, to me at least.

10. On p16, it would be natural to have a new paragraph between "30 minutes." and "Force-extension".

11. P16 - "The distance between the beads decreased" - the experimenters decreased this distance, correct, rather than it being an autonomous process? The phrasing is a bit unclear.

12. Bottom of p16 - define "overlapping region".

13. Equation 1 of the SI - would it make sense to index by window as well as by base?

14. There are a couple of missing refernces ([?]) in the SI.

15. Why is RNA disucssed at all in the SI? It's irrelevant to the main text.

16. The dot-paren labels on the x axis of some of the panels in Supplmentary Figure 2 are unreadable.

17. Supplementary note 3: the table on p16 is hard to interpret. Why all the empty boxes? Shouldn't the first box be x_{11}?

18. Supplementary figures 7-10: The caption needs to describe the figure, you can't just reference back to the text.

19. P30 of SI: previously, using the VEC pool to run a correlative analysis caused spurious apparent correlations. Is that not a problem here?

20. Captions to Figueres S26-S31: Ideally the first sentence would tell us what differentiates these figures from each other (I believe it is the time incubated before the gel was run).

21. Is there any explanation for the different sizes reported in Supplementary Table 6?

22. Supplementary note 15 - what does the relative trace length mean? WHy are they not all the same?

(Remarks on code availability)

Reviewer #2

(Remarks to the Author)
Noteworthy Results

This manuscript presents a framework for optimizing DNA origami scaffold sequences using four metrics that predict potential scaffold-staple mis-binding events. The authors demonstrate that their framework can identify scaffold sequences that improve folding yields in DNA origami nanostructures. Additionally, they show that structures folded with better-performing sequences exhibit less variability in mechanical response when tested with optical tweezers.

Significance and Originality

The optimization of scaffold sequences for DNA origami represents an important contribution to the field. The multi-objective approach to sequence design is novel and addresses a significant challenge in DNA origami fabrication. However, the work needs to be better contextualized with respect to prior studies:

1. Engelhardt et al. 2019 ACS Nano compared origami structures folded from de Bruijn sequences versus M13 scaffolds and found no significant differences in folding yield. The current study shows mixed results where T1 (de Bruijn) outperforms T2 (biological), but R2 (biological) outperforms R1 (de Bruijn). This apparent contradiction needs clarification.
2. The results should be discussed in relation to Aksel et al. 2024 Proc Natl. Acad. Sci., which suggests that folding accuracy depends on balancing hybridization energy with loop closure energy.

Data Analysis and Evidence

The experimental validation raises several concerns:
1. The gel electrophoresis images show significant smearing and higher-order bands in both scaffold and origami lanes. It's unclear whether folding issues stem from scaffold sequence or from scaffold quality problems.
2. The selection of the poorly-ranked T2 scaffold for testing seems inconsistent with the framework's predictions and requires explanation.
3. The statistical validity of the AFM analysis is questionable given the small sample size (~100 particles) compared to what gel analysis would provide. Was gel quantification performed to correlate with AFM findings?
4. Testing was limited to 2D origami structures, which typically fold more easily than 3D designs. Validation with more complex structures would strengthen the claims.

Methodology

The methodology appears sound but lacks sufficient detail in the main text:

1. The construction of the Pareto set is inadequately described. While Note 2 in the supplement details metric computation and Note 3 describes scaffold selection from the Pareto front, the method for determining points in the Pareto set is not clearly explained.
2. The synthesis of ssDNA scaffolds appears in the Results section but would be more appropriate in Methods.

Reproducibility

The 60-page supplement contains critical information that should appear in the main text:
1. Results from the 14-origami test set of previously published designs provide important context.
2. Gel electrophoresis data crucial for validating folding accuracy is relegated to the supplement.
3. Inconsistent naming schemes between figures and supplement make following the methodology difficult.

Figure 3d is difficult to interpret and appears redundant with Figure 3b, obscuring rather than clarifying the results.

Conclusion

This paper addresses an important problem in DNA origami design and presents a novel framework with promising experimental validation. However, significant reorganization is needed to improve clarity, and experimental concerns should be addressed before publication. The authors should:

1. Move critical information from the supplement to the main text
2. Address the relationship with previous work more thoroughly
3. Clarify the experimental validation issues
4. Provide better details on methodology and implementation

With these improvements, this work could make a valuable contribution to the field of DNA nanotechnology.

(Remarks on code availability)

Reviewer #3

(Remarks to the Author)
attached

(Remarks on code availability)
overrall the code seems to provide comprehensive instructions. However, I did not test the code due to time limitation.

Reviewer #4

(Remarks to the Author)
I co-reviewed this manuscript with one of the reviewers who provided the listed reports. This is part of the Nature Communications initiative to facilitate training in peer review and to provide appropriate recognition for Early Career Researchers who co-review manuscripts.

(Remarks on code availability)

Version 1:

Reviewer comments:

Reviewer #1

(Remarks to the Author)
Shirt-Ediss et al have edited their manuscript in response to my comments, and those of other reviewers. Most importantly, they have addressed my central criticism by including data for 2 new designs, which support the original story. Scientifially, therefore, the case is made for publication.

I am less convinced by the responses to my more minor comments. Sometimes an explanation is given, but something is not added to the text to explain the issue to other readers. See comments 2c,d,e, 2 (note, the numbering is off), 19, 22. In other cases, a explanation in the response seems a bit contradictory, even if the edited text is fine (see below). Although the response to these comments should probably not preclude publication, some may be worth reconsidering.

Minor points
1. How is the image in Fig 1A generated?

2. I note that the square images in the 6-helix bundle AFM images could be arranged more neatly, with the same dimensions and alignment.

3. 2a - while the text added to the manscript is ok, the argument that it is "obviously wrong" to use worse case scenarios for M1 and M2 surely could equally be applied to M3 and M4?

4. One does not need to sample from nupack to get *all* the sequence averaged parameters, including loop closing terms - these can all be directly cacluated from their definition in the supporting literature.

5. It is still unclear to me what information the coloured boxes in Fig 1 convey beyond the fact that a system consists of sequences and scaffold. What do we learn from them? I don't think the authors use them to illustrate the idea that the same scaffold can use different sequences, for example. One ends up looking for meaning in the boxes that doesn't seem to be there.

6. Previous comment 16 - how does the (unreadable) dot parenthesis notation help "the idea that the figure conveys" (the profiles are similar)? If it doesn't, why include it?

7. Previous comment 19 - I accept that the core function is not inhibited, but what value do these correlation plots provide? They do not directly contribute to the core function, unless I misunderstand.

8. Previous comment 22 - I couldn't find a definition of "Relative trace length" in the text itself.

(Remarks on code availability)

Reviewer #2

(Remarks to the Author)

The revisions are satisfactory. Aside from the following points, the manuscript is acceptable for publication.

1. Please define technical terms before they are presented in the main text (e.g., the Pareto set should be defined before it is used in the introduction, and the MCDM methods should be defined before they are used). There are additional places where the text could be simplified. For example, on page 6, the sentence about non-designed binding regions would be clearer if rephrased to directly state that random scaffolds are unlikely to contain repeated or complementary segments.

2. The authors have addressed previous concerns by adding experimental validation with a tetrahedron and 6-helix bundle. However, it should be noted that these wireframe designs are structurally similar to 2D shapes in terms of cross-section and folding difficulty. Future studies would benefit from including multilayer 3D origami structures, such as brick- or cube-like designs, of which there are numerous examples in the literature. This represents a more stringent test of the framework's predictive power for complex 3D assemblies.

(Remarks on code availability)

Reviewer #3

(Remarks to the Author)
I carefully read the revised manuscript, the reviewers' comments and the authors' responses. I had only minor comments for the original manuscript and all of those were satisfactory addressed. However, as for the original submission, I am not convinced that this manuscript is suitable for publication in Nature Communications.
While the work is of high quality, my hesitation to recommend it for the publication in Nature Communication comes from my, perhaps subjective, interpretation of the journal's aim and scope which states that "Papers published by the journal aim to represent important advances of significance to specialists within each field". I am having difficulty identifying "important advances of significance" in this work.
The work rather convincingly demonstrates that scaffolds with lower degree of off-target integrations improve yields in DO folding process. The authors also provide tools to compare the extend of off-target binding for different scaffold sequences. However, it is difficult for me to think of realistic scenario in which one would use those tools in practice for scaffold sequence optimization/selection*.
On the other hand, I agree with the authors that their results could be potentially useful for establishing more complete DNA origami design pipeline and I could support publication of this work in more specialized journal, e.g., Nucleic Acids Research.

-----
* I have been using DNA origami (DO) technique for over 15 years (folding origami since 2007) and while my research group focus is not in the field of structural DNA nanotechnology we utilizes the DO technique extensively. We care a lot about yields of DO assembly, still, minimization of off-target interaction is not a problem that we are concerned about in our DO design/fabrication/characterization pipeline. We (as probably vast majority of researchers using DO) use commercially available scaffolds. If our DO yields are not satisfactory, we optimize folding condition and, since recently, staple routing (Aksel et al). After such optimization, all our DNA origami designs (2D, 3D, twisted, bend, honeycomb and square lattice) fold well enough. This workflow is also very common in other labs, and I doubt that this work would change it, i.e., most of researchers will not optimize scaffold sequence as it is rather challenging from scaffold production point of view.

(Remarks on code availability)

Reviewer #4

(Remarks to the Author)
I co-reviewed this manuscript with one of the reviewers who provided the listed reports. This is part of the Nature Communications initiative to facilitate training in peer review and to provide appropriate recognition for Early Career Researchers who co-review manuscripts.

(Remarks on code availability)

Version 2:

Reviewer comments:

Reviewer #1

(Remarks to the Author)
The science is sound and while there are some things that I would phrase/present a little differently, this is the author's choice. I am happy to proceed to publication.

(Remarks on code availability)

Reviewer #2

(Remarks to the Author)
I am almost ready to recommend accepting the paper, pending one last change in response to my previous review item ("2. The authors have addressed previous concerns by adding experimental validation with a tetrahedron and 6-helix bundle. However, it should be noted that these wireframe designs are structurally similar to 2D shapes in terms of cross-section and folding difficulty.")

The authors' response is technically accurate—the 6-helix bundle (6HB) does indeed have a solid cross-section based on hexagonal lattice packing. However, this framing sidesteps the substantive point of the original concern. 6HBs are among the most reliably folding structures in the DNA origami repertoire, with decades of successful implementations across dozens studies. Their inclusion, while appropriate for initial validation, does not meaningfully stress-test the predictive framework against the kinetic and thermodynamic challenges that plague larger multilayer assemblies.

The authors themselves acknowledge that dense 3D structures present "additional challenges" due to fold-order dependencies and steric hindrance—precisely the complexities where predictive tools would be most valuable to the field.

The rebuttal demonstrates the authors understand this distinction, yet the revised manuscript does not reflect this nuance. A brief acknowledgment in the Discussion that validation on larger multilayer structures with significant fold-order constraints remains an open direction would strengthen the paper and better serve readers.

(Remarks on code availability)

Reviewer #4

(Remarks to the Author)
I co-reviewed this manuscript with one of the reviewers who provided the listed reports. This is part of the Nature Communications initiative to facilitate training in peer review and to provide appropriate recognition for Early Career Researchers who co-review manuscripts.

(Remarks on code availability)

Open Access This Peer Review File is licensed under a Creative Commons Attribution 4.0 International License, which permits use, sharing, adaptation, distribution and reproduction in any medium or format, as long as you give appropriate credit to the original author(s) and the source, provide a link to the Creative Commons license, and indicate if changes were made.
In cases where reviewers are anonymous, credit should be given to 'Anonymous Referee' and the source.

The images or other third party material in this Peer Review File are included in the article’s Creative Commons license, unless indicated otherwise in a credit line to the material. If material is not included in the article’s Creative Commons license and your intended use is not permitted by statutory regulation or exceeds the permitted use, you will need to obtain permission directly from the copyright holder.
To view a copy of this license, visit https://creativecommons.org/licenses/by/4.0/

Response to Reviewers - Reviewer #1

We thank the reviewer for having read in detail our paper and for making very valuable suggestions, which have substantially improved the paper. We have provided an annotated pdf with all the changes made to the paper but please find below a point-by-point response with review comments in blue, our response in black and any parts of the paper added or removed in green:

Shirt-Ediss et al. present work in which several metrics are proposed for assessing the likely success of DNA origami folding. These metrics are simplified estimates of the off-target staple binding to the scaffold, scaffold-scaffold binding, staple-staple binding and self-binding of a single staple. The authors discuss how variable these metrics are for a range of origami designs and a range of methods for selecting the scaffold (extracts from biological vectors, random, and a "De Bruijn pool" in which sequences of length greater than or equal to k, with k=5 or 6, are never repeated). They find that biological vectors produce the largest variance over these metrics, and also that they tend to score worse on the first two metrics at least. The authors suggest that the low variance of random and De Bruijn pools suggest that sequence selection is not necessary in those cases.

The authors then tested their metrics by designing 3 variants (optimized De Bruijn, optimized biological vector and pathological biological vector) of 2 origami shapes (triangle and rectangle). In each case, the De Bruijn sequence is predicted to outperform the optimized biological vector, which is predicted to outperform the pathological biological vector. The authors perform folding experiments and demonstrate that this behaviour is observed, except for the fact that the optimized biological vector slightly out-performs the optimized De Bruijn sequence. Finally, force-extension data is reported for the three triangular designs, with traces being more uniform for the De Bruijn sequence and less uniform for the pathological biological vector, suggesting a more disparate ensemble of folded structures.

The authors address an interesting question appropriate for the broad readership of Nature Communications. The metrics are an interesting attempt to simplify the problem of identifying off-target interactions in origami assembly. The theory is combined with two types of experiment to assess its predictive power. The manuscript is well written and generally easy to follow. On that basis I am minded to support the progress of the work towards publication. My main concern is the degree to which the theoretical predictions have been robustly tested.

Main concern:
1. The authors have only assessed their system using the folding of two origami shapes, with three designs each, and by looking at the force-extension properties of three designs of the same shape. The results are encouraging, but this is a fairly small sample - we certainly cannot, at the moment, be confident that the metrics work in general. It is worth noting that the two biological vectors considered have been selected from the pareto front and reverse front of the metrics -- in some sense, providing the easiest challenge for discrimination. It is less clear how reliable the authors approach would be in improving folding relative to a typical biological vector.

In our revised paper, we have added full experimental results for a further two 3D origamis: a tetrahedron (in 3 variants) and a 6-helix bundle (in 3 variants). These extra experiments confirm our previous finding that origamis with high off-target interactions largely fail to fold.

For the second part of the reviewer's question, we don't see there is such thing as a 'typical' biological vector. Given a large vector like Lambda DNA and tasked with identifying a sequence region to amplify for the origami scaffold strand, it could be argued that all sequence regions of Lambda could be chosen at the same probability. A good or a bad sequence would have the same likelihood - there is not a sequence region that would be 'typically' chosen. By selecting the worst vector from the reverse front, and the best vector from the Pareto front, we are simply showing what range of folding behaviour could be observed if an experimenter made a random choice of Lambda DNA sequence region for origami scaffold.

Significant comments:
2. The motivation for some aspects of the model design were a bit unclear to me.

(a) Why do the authors use totals for M1 and M2, but only worst single case for M3 and M4?

We thank the reviewer for this observation. If M3 and M4 were totals like M1 and M2, then being summations, these totals could mask some potentially strong and disruptive off-target staple-staple (M3) and staple hairpins (M4).

A strong staple-staple co-fold, or a strong staple hairpin could potentially disrupt the folding of an origami design if the staple(s) involved was at a key place, like joining two separate pieces of the structure together (like at the seam of a triangle).

Instead, the decision was taken to make M3 and M4 represent the worst case, such that strong staple-staple and staple hairpins were definitely avoided (rather than becoming masked in an aggregate summation).

Conversely, using worst-case bindings would not make sense for M1 or M2, mainly due to the length of the scaffold strand. For example, in M1, a scaffold with a single -8kcal/mol off-target region would be scored the same as a scaffold with 1000 -8kcal/mol off-target regions: obviously incorrect. Similarly in M2, a scaffold with a single scaffold-scaffold binding of -20kcal/mol would be scored as worse than another scaffold with a 1000 binding regions equal to or less than -18 kcal/mol, again obviously incorrect.

However, this is an important observation by the reviewer, and a distinction we were also mindful of during the design of metrics. Finally, we made an educated choice. However, as mentioned in the discussion, we are aware that future extensions of the work could also systematically investigate for example, using totals for M3 and M4 instead (and tested experimentally).

We have added the following text to the Supplementary Material, for Metric 3:

“Metrics 3 and 4 are calculated as worst cases, instead of summations, to definitely avoid very strong staple inter and intra- folds, that could potentially disrupt the folding of an origami design if the staple involved was at a key place, like joining two separate pieces of the structure together (like at the seam of a triangle). A summation is not guaranteed to avoid these cases.”

(b) Why use a sequence-averaged model? Is the extra cost of including the specific base pair free energies (see https://pubmed.ncbi.nlm.nih.gov/15139820/) significant?

Thanks to the reviewer for the question. We used a sequence-averaged model because it's fast. It just does binary comparisons and it must do millions of calculations for different binding permutations.

We have added this sentence at the beginning of Supplementary Note 1, to be clear: “Identifying off-target reaction sites is compute heavy, and so a fast sequence-averaged energy model is used to identify and calculate the (approximate) free energy of on-target and off-target binding sites in metrics M_{1} and M_{2}”

For the second part of the reviewer question: As shown in the SI, the sequence-averaged model when compared to NUPACK forms a comparable energy landscape for DNA-DNA bindings. True, a simple Nearest-Neighbour sequence-dependent energy model could be introduced, but it would only add algorithm complexity (and with it, an increased possibility for calculation errors) without really improving prediction accuracy for DNA.

RNA, on the other hand, is a different story. We already explicitly suggest in the discussion that a sequence-averaged model is not accurate for RNA and a sequence dependent model needs to be developed in this case.

(c) When using the sequence-averaged model, why not just directly average the published parameters (see https://pubmed.ncbi.nlm.nih.gov/15139820/) rather than try to reverse engineer them by averaging over nupack outputs? Are the results obtained significantly different from taking this approach?

Again, thank you for the question. The parameters for the sequence-averaged model were sampled from NUPACK to remain consistent with M3 and M4 that both use NUPACK directly. Sampling from NUPACK also gets parameters for RNA, and includes averaged energy corrections for loops where the sequence at the beginning and end of the loop contribute to the loop energy.

However we agree that another approach would be to average the published parameters, as the reviewer suggests, and just use the Jacobson-Stockmayer approximation for symmetric loop energies.

(d) When the authors say (in the SI) that the absolute value of dGs were used, is it true that all dGs entering in to the metrics are negative? Otherwise there is a slightly strange effect where a really positive free energy (which is irrelevant to folding) would be interpreted as equivalent to really stron gbinding?

Thanks for this technical observation. We note that:

- In Metric 1, dG_offtarget is clipped and cannot exceed 0, thus the reviewer comment does not apply here.
- In Metric 2, yes, it is theoretically possible that some permutations of the scaffold with itself result in positive dG values. However, these can be expected to be rare at the 37C calculation temperature. The large majority of permutations will have zero or negative dG values. M2 would only have an erroneous calculation if the number of positive dG values were significant.
- In Metric 3, the minimum free energy dG of co-fold over all staple pairs is calculated. There only has to be one negative dG co-fold with magnitude greater than greatest positive dG, for metric to be valid. This condition should be easily satisfied.
- Metric 4: Same as M3. There only has to be one negative MFE dG of a hairpin greater in magnitude than largest positive dG for the metric to be valid. This condition should be easily satisfied.

In summary, the reviewer does raise a valid point regarding a pathological technical possibility.

In practice, however, metric mis-calculation via positive dG values is unlikely to be significant, especially at the (relatively low) calculation temperatures used.

(e) In general, the authors don't try to weight the metrics relative to one another in a careful way. Nonetheless, it is worth noting that it would be possible to do a more careful alignment of M3 and M4. M4 is an intramolecular folding and M3 is an intermolecular association, with the strands typically at concentrations of 10s of nanomolar. The same standard free energy dG0 for both corresponds to a very different likelihood of problems. For M4, the likelihood of a structure relative to the unfolded state is exp(-dG0/RT). For M3, the likelood of a duplex relative to separated strands is (very roughly) [concentration of strands]/[reference concentration] * exp(-dG0). Since the reference concentration of Nupack is 55M (see the original Dirks et al paper), the ratio [concentration of strands]/[reference concentration] is of order 10^9. This is a massive difference that really should feature in the metrics. In practice, it won't change the pareto front but would it change some of the analysis that the auhtors do? To a lesser extent this is also true of the scaffold self-folding versus the staple mis-bonding, but the authors are not carefully calculating looping costs for the scaffold anyway, so the direct comparison can't be made.

The beauty of calculating a Pareto front, unlike when calculating a single objective function, is that the dimensions of the objective space do not need to be correctly weighted relative to each other. Dimensions can be completely different magnitudes, and even in different units, and the resulting Pareto solution set is not affected.

In our study, there is no requirement to correctly weight M3 and M4 relative to each other. Also, in the multi-criteria decision making calculations we do, the metrics are

normalised, but only relative to themselves (Eq 13, SI). Therefore, their absolute magnitudes are not significant here either.

However, we thank the reviewer for the calculations nevertheless, which could be useful if M3 and M4 were combined into a single metric in a future extension to our study.

2. The authors present an analysis of the inaccuracy of using their sequence-averaged description, but not the cost of ignoring asymmetric loops. Is it possible to estimate that, for small systems at least?

We thank the reviewer for this relevant question. However, this is a non-trivial point to address.

The enumeration of all possible asymmetric loops in M1 and M2 would require significantly more computational resource (and the implementation of more complex dynamic programming algorithms) as compared to the enumeration of symmetric loops that just requires sequence alignment. This is why we stopped at symmetric loops, which is already a significant improvement over not considering loops at all.

Calculating the true cost of asymmetric loops in M1 and M2 would be another project, even to estimate for small systems.

We emphasise that, in addition to accuracy, a key requirement for M1 and M2 calculation is computational efficiency. We clearly state in the paper that our approach is heuristic. The metrics are intended to be fast-to-calculate heuristics of good folding, but not absolute indicators of it. We consider that including symmetric loops gives a good enough folding heuristic, without going into the thorny details of asymmetries.

We draw to the reviewers attention that M3 and M4 do already incorporate asymmetric loops, as they use NUPACK to compute MFE structures.

3. The main text contains quite a lot of discussion of data that I think is only presented in the SI (see p6, p8, p12). This makes it quite hard to follow some of the arguments - data relegated to the SI should, ideally, not require a detailed discussion.

The reviewer is correct - some data is only presented in the SI. However, this was out of necessity. This paper was an ambitious long-term study, spanning many years – and a lot of work. It involved both a substantial computational and experimental component. Unfortunately, the depth of the study means that some discussion of background results needs to take place in the SI.

We do state summaries of the detailed computational and experimental results in the paper to save space for the main experimental results and the main message of the paper.

4. Was the classification of structures performed by hand? Are there details on the

systematic approach used to classify? What were the original rules followed by those in Newcastle?

The classification of assemblies was performed by hand. As mentioned in the paragraph “Notes on blind experimental protocol and independent replication”, examples of “semi-folded” and “mis-folded” categories (Supplementary Note 17) were shared by Newcastle to allow a correct quantification of assemblies. Bonn’s team classified the DNA assemblies from AFM images following the guidelines reported in Supplementary Note 17.

The sentence “Examples of “semi-folded” and “mis-folded” categories were shared by Newcastle to allow a correct quantification of assemblies (see Supplementary Note 17)” has been moved from Results (paragraph “Notes on blind experimental protocol and independent replication”) to Methods (in “DNA origami: folding reactions, purifications and AFM imaging conducted at University of Bonn, Germany.”).

Minor comments:
5. What do the coloured blocks in Fig 1 represent?

This is explained in the Figure 1 caption: “Each variant, denoted as a pair of coloured blocks, consists of a unique scaffold sequence along with the Watson-Crick complementary staple sequences that pin the scaffold strand into a specific rotation.”

The coloured blocks are just a simple visual shorthand of the origami sequence set. The same origami shape can be made from many different coloured block pairs: different scaffold sequences with complementary staples, or same scaffold sequences at different rotations (giving different staple sequences for each rotation).

6. What is the motivation for the different experimental protocols used for different systems (see first column, p15, and Supplementary Table 5).

Double-stranded DNA scaffold templates (6 different sequences) were amplified by polymerase chain reaction (PCR) using 6 different forward and reverse primers’ pairs (Supplementary Table 4) complementary to each template and characterized by specific melting temperatures.

To ensure a successful PCR for each template, the annealing temperature, annealing duration, number of cycles and template amount were optimized (Supplementary Table 5). For high melting temperature primer pairs, a 2-step PCR was run combining annealing and extension (Lambda Bear and gBlocks Deer in Supplementary Table 5).

In Methods (par. “Double-stranded DNA amplification, purification, and agarose gel electrophoresis”), the main text was improved accordingly from “The cycling conditions and the template quantity (ng in 50μL) for each variant are reported in Supplementary Table 5.” to “To ensure a successful PCR for each template, the annealing temperature, annealing duration, number of cycles and template amount were optimised (Supplementary Tables 5 & 7). For high melting temperature primer

pairs, a 2-step PCR was run combining annealing and extension (Lambda T3 and gBlocks T1 in Supplementary Table 5).”

7. Perhaps I missed it, but I was unsure how the scaffold strand for the De Bruijn system was obtained

The scaffold strands for the DBS were obtained from the gBlocks Gene fragments (see Methods, paragraph “Double-stranded DNA amplification, purification, and agarose gel electrophoresis”). The sentence “Double-stranded gBlocks Gene Fragments and Lambda DNA were amplified…” was improved accordingly as follows: “Double-stranded gBlocks Gene Fragments (De Bruijn sequences) and Lambda DNA were amplified...”.

8. On p12, the authors discuss "received wisdom" that any scaffold will fold. Are there any sources to back this up?

We thank the reviewer for this question. We picked up this 'received wisdom' view from talking with people at conferences about origami assembly, and from conference presentations that talk about an origami “always selecting” the minimum free energy structure in the end. But little is written about this in the literature.

Therefore, to take a softer approach, we have now removed the following paragraph from the discussion: “This view challenges received wisdom (15, 24) in the DNA nanotechnology community that, an origami will always self-select the minimum free energy structure (with all staples bound in their designed complemen- tary positions) from any initial scaffold/staple strand cocktail, for reasonably heterogeneous sequences.”

We took the opportunity remove a similar paragraph from the introduction: "because of the perceived robustness of the DNA origami folding process[15,24]."

We made some small textual changes to the introduction to make it flow again. Now we simply say that off-target reactions are an overlooked aspect worth also considering.

9. The meaning of the "threshold (caption of fig 14) was not that obvious from the main text, to me at least.

The reviewer must be referring to Fig. 6 in the reviewed version (now figure 7 in the new text), where we indicate in the caption that we used a 0.14 threshold. We did not explicitly write that this is a threshold on the non-uniformity metric (NUM), so we now specify it as requested. In addition, the reviewer may be wondering about the specific value we used (0.14). This is in fact somewhat arbitrary, but as can be seen in Supplementary Fig. 48d, the choice of threshold does not affect the qualitative differences between the different designs.

Thus, the caption on figure 7 on the main text now reads: “The non-uniformity threshold for the clusters displayed in this figure is set to 0.14, but the observed

clustering is qualitatively insensitive to the choice of threshold (Supplementary Figure 48d)".

10. On p16, it would be natural to have a new paragraph between "30 minutes." and "Force-extension".

The reviewer meant page 17, and that is correct; there should be a new paragraph after "30 minutes", and "Force-extension curves acquisition." Should be in italic as it its a subtitle. This has now been corrected in the new version, bottom of first column on page 17 of the main text.

11. P16 - "The distance between the beads decreased" - the experimenters decreased this distance, correct, rather than it being an autonomous process? The phrasing is a bit unclear.

Also on page 17, and a fair point, which has now been corrected to "If the tether remained intact, the distance between the beads was decreased and then increased again…"

12. Bottom of p16 - define "overlapping region".

The reviewer is correct that this is confusing and indeed, it is not needed. The text now simply reads "For each pair, we low-pass filtered the data to10 Hz"

13. Equation 1 of the SI - would it make sense to index by window as well as by base?

We thank the reviewer for the comment. Equation 1 of the SI is now indexed by window duplex index d as well.

14. There are a couple of missing references ([?]) in the SI.

We thank the reviewer for the comment. These two missing references have been added.

15. Why is RNA discussed at all in the SI? It's irrelevant to the main text.

The main text does mention RNA in the last paragraph of the discussion, when discussed how the approach made me extended to RNA objects.

Now, in the last paragraph of the Discussion in the main paper, we have added a link to Supplementary Note 1 where RNA energies are discussed, so this section does not seem irrelevant to the main text.

16. The dot-paren labels on the x axis of some of the panels in Supplementary Figure 2 are unreadable.

Unfortunately, the scale of the right hand plots in Supplementary Figure 2 mean the X-axis is highly compressed. However, the x-axis is not necessary to read literally in this case. The idea that the figure conveys is the overall similarity between the sequence averaged energy model, and a detailed energy landscape created by NUPACK over large sliding window sizes. We have not modified this figure.

17. Supplementary note 3: the table on p16 is hard to interpret. Why all the empty boxes? Shouldn't the first box be x_{11}?

We thank the reviewer for the comment. The table has now been filled with numbered variables (x11, x21 etc) and is more clear. Note that the table simply lists all alternatives on the Pareto front in matrix form.

18. Supplementary figures 7-10: The caption needs to describe the figure, you can't just reference back to the text.

We thank the reviewer for the comment. We removed "See text for description" from these figure captions. Instead, we added a descriptive caption: "(a) density of off-target sites; (b) how much metric scores can be optimised, see text for definition of r; (c) direct comparison of data in panel (a)."

19. P30 of SI: previously, using the VEC pool to run a correlative analysis caused spurious apparent correlations. Is that not a problem here?

We thank the reviewer for this pertinent question.

When spurious apparent correlations exist, the mass of points in objective space becomes more elongated and fragmented (like in Supplementary Figure 5). In effect, the objective space reduces in dimensionality because some of the objectives are related.

However, this does not inhibit sequence selection from performing its core function.

From a computational perspective, a Pareto set is still defined, and there still exists a large relative difference between good solutions on the Pareto front, and "bad" solutions on the Reverse front (i.e. off-target bindings can be decreased by a large relative percentage).

Our experimental results show that bad-folding origamis could still be distinguished from good-folding ones despite these correlations.

20. Captions to Figures S26-S31: Ideally the first sentence would tell us what differentiates these figures from each other (I believe it is the time incubated before the gel was run).

The first sentences of the figure's captions were changed as follows.

Supplementary Figure 32: Gel electrophoresis pre-screening of triangle DNA origami variants (T2 Lion, T3 Bear, T1 Deer) assembled at three different temperature conditions (isothermal annealing with or without initial denaturation, and fast ramp).

Supplementary Figure 33: Gel electrophoresis pre-screening of triangle DNA origami variants (T2 Lion, T3 Bear, T1 Deer) assembled at four different temperature conditions (isothermal annealing with or without initial denaturation, fast and slow ramps).

Supplementary Figure 34: Gel electrophoresis pre-screening of triangle DNA origami variants (T2 Lion, T3 Bear, T1 Deer) assembled at four different temperature conditions (isothermal annealing with or without initial denaturation, fast and slow ramps), and kept at 4 °C for 4 or 5 days.

Supplementary Figure 35: Gel electrophoresis pre-screening of rectangle DNA origami variants (R2 Lamb, R3 Mole, R1 Goat) assembled at three different temperature conditions (isothermal annealing with or without initial denaturation, and fast ramp).

Supplementary Figure 36: Gel electrophoresis pre-screening of rectangle DNA origami variants (R2 Lamb, R3 Mole, R1 Goat) assembled at four different temperature conditions (isothermal annealing with or without initial denaturation, fast and slow ramps).
Supplementary Figure 37: Gel electrophoresis pre-screening of rectangle DNA origami variants (R2 Lamb, R3 Mole, R1 Goat) assembled at four different temperature conditions (isothermal annealing with or without initial denaturation, fast and slow ramps), and kept at 4 °C for 4 or 5 days.

21. Is there any explanation for the different sizes reported in Supplementary Table 6?

We added this rationale to the caption of Supplementary Table 8:

To rationalise the difference in measurements: at University of Bonn, the High-Resolution AFM imaging was conducted with a JPK NanoWizard®3 Ultra AFM (piezo excitation) and using ultra short cantilevers (USC-F0.3-k0.3) which are dedicated to High-Speed scanning. The AFM tip has different geometry and characteristics (cone shaped, HDC/DLC material and dimension) compared to the BioLever Mini probe (3-sided shaped, silicon) used at Newcastle University (Cypher ES AFM with photothermal excitation). Thus, the topography of the imaged objects is affected by the shape and lateral dimensions of the AFM tips (Fuentes-Perez M.E. et al., 2013; Kolbeck P.J. et al., 2023).

Fuentes-Perez M.E., et al., 2013. AFM volumetric methods for the characterization of proteins and nucleic acids. Methods, 60, 2, 113-121.

Kolbeck P.J., et al., 2023. DNA origami fiducial for accurate 3D atomic force microscopy imaging. Nano Letters, 23, 4, 1236–1243.

22. Supplementary note 15 - what does the relative trace length mean? WHY are they not all the same?

“Relative trace length” means trace length divided by scaffold length. The reason they are not all the same is that in some experiments the tether breaks before the origami fully disassembles.

Response to Reviewers - Reviewer #2

We thank the reviewer for their time reading our paper and for making helpful, detailed, comments, which have helped us improve it. We provide an annotated pdf with all the changes made to the paper but please find below a point-by-point response with review comments in blue, our response in black and any parts of the paper added or removed in green:

Noteworthy Results

This manuscript presents a framework for optimizing DNA origami scaffold sequences using four metrics that predict potential scaffold-staple mis-binding events. The authors demonstrate that their framework can identify scaffold sequences that improve folding yields in DNA origami nanostructures. Additionally, they show that structures folded with better-performing sequences exhibit less variability in mechanical response when tested with optical tweezers.

Significance and Originality

The optimization of scaffold sequences for DNA origami represents an important contribution to the field. The multi-objective approach to sequence design is novel and addresses a significant challenge in DNA origami fabrication. However, the work needs to be better contextualized with respect to prior studies:

1. Engelhardt et al. 2019 ACS Nano compared origami structures folded from de Bruijn sequences versus M13 scaffolds and found no significant differences in folding yield. The current study shows mixed results where T1 (de Bruijn) outperforms T2 (biological), but R2 (biological) outperforms R1 (de Bruijn). This apparent contradiction needs clarification.

We thank the reviewer for encouraging more comparison with previous findings.

Engelhardt et al. compare the folding of a 42 helix bundle using a DeBruijn scaffold and an M13 scaffold.

To answer the first part of the reviewer comment: In these particular experiments, Engelhardt et al. did not observe folding yield differences between DeBruijn or M13 scaffold. However, this does not imply that, in general, *any* biological sequence will have the same folding yield as a DeBruijn sequence. They tested only the M13 scaffold, and only with a single origami shape.

In our study, we found that certain choices for biological sequences (mainly, certain sections of the lambda vector) were bad folders, whereas other choices for biological sequences were good folders. We surveyed a much wider range of biological sequences, drawn from multiple biological sources, which was not performed in Engelhardt et al. and we also tested 4 different origami shapes.

To answer the second part of the reviewer comment: The biological sequence rectangle R2 (with more off-targets) does indeed outperform the DeBruijn sequence rectangle R1 (with less off-targets). What are the reasons for that?

In our opinion, there could be multiple factors at play here.
Factor 1: R2 is the best biological sequence, which has more off-targets than R1, but because it's a 'good' biological sequence, the margin is not huge (unlike the difference between R2 and R3), particularly for number of off-target scaffold-scaffold bindings (metric M2).

Factor 2: DNA synthesis and purification may be an outside factor complicating the fair comparison of DBS to biological. (Although independent replication of results at University of Bonn in the SI gave the same outcomes)

Factor 3: It could be that minimising off-target interactions may not be the whole story for successful folding in some cases. This was already mentioned in the Discussion section.

Important to note is that our approach does reliably differentiate when the scaffold source is biological e.g. T2/T3, and R2/R3. And that is a main use of our algorithm: most labs will be using biological sequences because of low price/easy availability and the algorithm can say what regions give good and bad folders.

We have added the following sentences to the Discussion section, adding to our existing comment on R2 folding better than R1. We added the text in red.

"The same anomaly was also observed for the rectangle origami: R2 folded with the highest AFM yield, followed by R1 and then R3. This anomaly, where R2 with more off-target reactions assembles at a higher yield than the de Bruijn sequence control R1 may point to the fact that there may exist other, hidden sequence design principles that can increase assembly yield in the case of specific origami shapes. But we also note that DNA synthesis and purification may be an extra complicating factor the fair comparison of DBS to biological sequences."

2. The results should be discussed in relation to Aksel et al. 2024 Proc Natl. Acad. Sci., which suggests that folding accuracy depends on balancing hybridization energy with loop closure energy.

Again, we thank the reviewer for encouraging more comparison with previous findings. The Aksel et al. paper was only a preprint at the time of submission, and so we did not include it.

We actually fully agree with the general statement made in Aksel et al. that "Accurate folding requires staple routes that balance the enthalpically favorable formation of staple–scaffold duplexes via hybridization with entropic penalties of scaffold loop-closures". This is intuitively correct: an origami is less likely to fold if staples all have very short legs (low dGhyb) and all make unnecessarily long scaffold loops (high dGloop) -- although co-operativity during folding can play a significant role too.

However, our approach diverges with that of Aksel et al. in important ways:

- We rigorously consider the thorny problem of **off-target reactions**, and determine optimal scaffold/staple sequences from a finite sequence pool **given** a fixed staple layout. Our algorithm is a sequence selector/designer.
- Aksel et al. don't consider off-target reactions at all, and determine the optimal staple layout **given** a fixed user-supplied scaffold sequence, plus some stochasticity. The Aksel et al. algorithm is a staple routing designer.

In summary, our study cannot be directly compared to Aksel et al. as their analysis is based at the level of staple routing, not the lower-level off-target reactions. We start from the premise that an acceptable staple routing exists for an origami, and then we design the scaffold sequence explicitly, taking into account the minimisation of off-target reactions.

In light of this, we have incorporated the Aksel 2024 paper in the discussion with this paragraph:
"Finally, our work may be used as part of a pipeline, as a final sequence design stage, after staple-routing algorithms [REF to Aksel et al.] (which do not take into account off-target reactions) have made an initial acceptable layout of staples."

We have also referenced the Aksel paper in the introduction, where we talk about studies that investigate factors affecting DNA origami assembly.

Side notes: Our opinion is that the Aksel et al. origami scoring function, although based on thermodynamics, leans on some quite questionable assumptions (which they also seem aware of). One is that off-target interactions are completely ignored. Another is that they calculate the loop closure energy of each staple **independently**, in the absence of all other staples (using a simple equilibrium binding model). However, it is well known that the DNA origami folding pathway is actually determined by **staple co-operativity** which significantly modifies loop entropy costs and staple melting temperatures as the structure kinetically folds up. Staples certainly do not bind independently and, in turn, it does not actually make sense to calculate staple melting temperatures by considering staples in isolation. However, generally, the Aksel et al. algorithm can be seen to design structures with well defined nucleation centres and fast nucleation fronts, which should indirectly assist co-operative binding and good folding.

Data Analysis and Evidence

The experimental validation raises several concerns:
1. The gel electrophoresis images show significant smearing and higher-order bands in both scaffold and origami lanes. It's unclear whether folding issues stem from scaffold sequence or from scaffold quality problems.

Noteborn and coll. (2021, Ref. 38) demonstrated the self-assembly of 4 different DNA origami using single stranded scaffolds obtained by PCR and subsequent T7 exonuclease digestion with no off-target degradation of sense DNA. Phosphorothioate protective modification at the 5’ end of the desired sense ssDNA strand inhibits the exonuclease digestion (from 5’ to 3’), while the non-required antisense strand is digested (digestion rate 5.3-5.7 bases/sec, Takahashi *et al.*,

2014). We considered the same strategy as reported in Methods. After the overnight digestion and purification, the single stranded DNA was analysed by gel electrophoresis. In the gel images (Supplementary Note 13), the digested samples (6) showed similar band pattern characterized by a higher band with the same migration distance of the template dsDNA bands, suggesting that a small quantity of dsDNA was not digested.

The faint smearing above the ssDNA bands was previously showed by others (Noteborn *et al*., 2021, Marimuthu *et al*., 2012, Kujau and Wolfl 1997) and related to different ssDNA conformations (Marimuthu *et al*., 2012). The electrophoretic analysis of the six ssDNA scaffolds also underlined the absence of lower (faster migration compared to the desired ssDNA) bands and smearing due to degraded sense ssDNA products, indicating no off-target degradation of sense DNA as previously reported (Noteborn *et al*., 2021).

To verify the single stranded scaffold sequence composition (sense ssDNA strands with phosphorothioate protective modification at the 5' end), Sanger sequencing from the 3' end (Veneziano *et al*., 2018) was performed using the same primers used for the dsDNA template amplification (please refer to "Synthesis of single-stranded DNA scaffold sequences" in Results and "Purified double-stranded DNA digestion using T7 exonuclease, purification, and agarose gel electrophoresis" in Methods). Two or more overlapping traces in the chromatograms representing different sequencing products were not identified.

Takahashi, S., *et al*. (2014). Real-time single-molecule observations of T7 exonuclease activity in a microflow channel. *Analytical Biochemistry* 457, 24-30.
Marimuthu, C., *et al*. (2012). Single-stranded DNA (ssDNA) production in DNA aptamer generation. *Analyst* 137, 1307.
Kujau, M.J. and Wolfl, S. (1997). Efficient preparation of single-stranded DNA for in vitro selection. *Molecular Biotechnology* 7, 33-335.
Veneziano, R. *et al*. (2018). *In vitro* synthesis of gene-length single-stranded DNA. *Scientific Reports* 8, 6548.

2. The selection of the poorly-ranked T2 scaffold for testing seems inconsistent with the framework's predictions and requires explanation.

We thank the reviewer for this observation. The reason that the SAW, KNEE and TOPSIS ranks of T2 (in the table of Figure 3b) are higher than for the other origami variants is because the T2 sequences were the very first to be ordered for our experiments. At this very early stage of the project, MCDM scores SAW, KNEE and TOPSIS were being normalised by the metric ranges across the extent of the Pareto Front only. Using this normalisation, T2 ranked as number 1 under these MCDM methods. Later, the MCDM normalisation was changed to use the metric ranges across the extent of the entire objective space. The other origamis were selected with this new normalisation. At this stage, however, the triangle could not be modified as the sequences had already been ordered and many hours of experimentation had been done.

Importantly, it should be noted that the T2 scaffold we used is not “poorly ranked”. It is still on the Pareto front (ranking of 36 out of 207 variants on the Pareto Front), and the Pareto Front itself is only 4% of all sequence variants scored.

The table below shows a comparison of the T2 we used, and the “optimal” T2 which could have been selected using the new MCDM normalisation scheme.

	M1	M2	M3	M4
T2 used	3033.89	1578.58	9.38	0.72
T2 optimal	2661.96	1367.57	8.71	1.28

The optimal T2 does have slightly less M1 and M2 off-target interactions, but the difference between the origamis is not huge.

The T2 we used shows:

- Other individuals on the Pareto Front can also fold well, not just the top ranked individuals by MCDM
- The MCDM normalisation scheme we eventually settled on is actually more appropriate than the initial scheme we tried, since it finds variants with lower metric scores on the Pareto front.

3. The statistical validity of the AFM analysis is questionable given the small sample size (~100 particles) compared to what gel analysis would provide. Was gel quantification performed to correlate with AFM findings?

The gel electrophoresis analysis was considered as a pre-screening through indicators such as band sharpness and migration distance (Wagenbauer K.F. *et al.*, 2017). This analysis can allow an initial estimation without providing in-depth structural information achievable through more sophisticated techniques (e.g., AFM) (Dey S. *et al.*, 2021). For this reason, the quantification was based on AFM images. As suggested, the size of the samples was increased (see Figure below), except for T3 variant characterized by a low deposition efficiency.
Wagenbauer K.F. *et al.* (2017). How we make DNA origami. *ChemBioChem* 18, 1873-1885.
Dey S. *et al.* (2021). DNA origami. Nature Reviews 1:13.

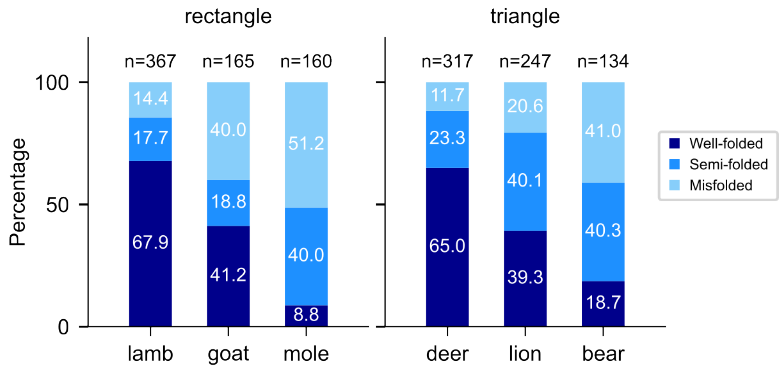

Updated manual quantification of origami objects on AFM images into "well-folded", "semi-folded" and "mis-folded" categories. The percent of origami objects in each category is shown.

4. Testing was limited to 2D origami structures, which typically fold more easily than 3D designs. Validation with more complex structures would strengthen the claims.

We ran extra experiments on two new 3D DNA origamis. 3D DNA wireframe tetrahedron variants and 6-helix bundle rod variants were designed *in silico* using our algorithm, synthesized, purified, and characterized by AFM imaging. Details were added in the main text and in the Supplementary Information. Both 3D origami nanostructures validated our claims.

Methodology

The methodology appears sound but lacks sufficient detail in the main text:

1. The construction of the Pareto set is inadequately described. While Note 2 in the supplement details metric computation and Note 3 describes scaffold selection from the Pareto front, the method for determining points in the Pareto set is not clearly explained.

We thank the reviewer for catching this - it will help people not familiar with multi-objective optimisation. We have added the following brief definition of a Pareto Front to the top of Supplementary Note 3:

"The Pareto front of optimal solutions in the objective space (defined by the metrics in Supplementary Note 2) is calculated as follows: Point A dominates Point B if A has no metric greater than B, and at least one metric smaller. The Pareto front is the collection of points that are not dominated by any other point in the objective space."

In the text, we also added a link to Supplementary Note 3, when talking about the Pareto front: "where a set (called the `Pareto front') of optimal trade-off sequence variants becomes defined (black dots, see Supplementary Note 3)"

2. The synthesis of ssDNA scaffolds appears in the Results section but would be more appropriate in Methods.

Some paragraphs in "Synthesis of single-stranded DNA scaffold sequences" (Results) were moved to Methods as suggested.

Reproducibility

The 60-page supplement contains critical information that should appear in the main

text:
1. Results from the 14-origami test set of previously published designs provide important context.

We thank the reviewer for recognising that the computational investigation of the 14 origami test provides important background context. Indeed, it was a lot of work, taking 1 week on an HPC cluster. However, we cannot provide more detail about these computations in the paper, simply because of the limited space available.

Instead, we save the paper space for the experimental results (which are the real acid tests of the algorithm). At the request of reviewers, we have also had to add more experiments to the paper, limiting space further.

We mention just the main conclusions of the computational investigation and we refer to Supplementary Note 4 where suitable in the text.

2. Gel electrophoresis data crucial for validating folding accuracy is relegated to the supplement.

We did include the main important gel electrophoresis data in Figures 4, 5, and (new figure) 6. Full gel electrophoresis data is in the supplement because (i) it shows the effect of different temperature ramps other than the fast temperature ramp we used and (ii) the gel data was not the main information source we used to validate folding accuracy – instead we counted objects on AFM images.

We followed this reasoning process:

The agarose gel electrophoresis (AGE) analysis (pre-screening) was conducted on samples folded following different temperature ramps (slow 5h 40 min and fast 1h 15min) or isothermal folding protocols with or without initial denaturation at 95°C.

The different temperature conditions and related results are mentioned in paragraph: “Triangle and rectangle DNA origami variants: folding and pre-screening by gel electrophoresis” (in Results).

Supplementary Note 14 includes gel images for each temperature condition (4). Based on the AGE pre-screening results, we selected only the fast ramp for further purification, characterisation by AFM (crucial data) and algorithm validation. AGE (fast ramp) was repeated and images were included in Figures 4 and 5, specifying in the captions “The assembly reaction was performed under a fast temperature ramp of 95°C to 20°C in 1h 15min (-0.1°C per cycle, each cycle 6 seconds)”. These gel electrophoresis data (fast ramp) were combined to the AFM images and included in the main text (Figure 4 and 5). All the other gel images (corresponding to not selected temperature conditions) were included in the Supplementary Information.

3. Inconsistent naming schemes between figures and supplement make following the methodology difficult.

We did not fully understand what the reviewer meant by “inconsistent naming schemes”. Our assumption was that it refers to the origami names.

Therefore, in the methods section of the paper, we have renamed the blinded origami names e.g. Deer, Lion, Bear etc. to the origami names used in the paper e.g. T1, T2, T3.

Further, In the Supplementary Material, we now give the T1/T2/T3 R1/R2/R3 origami name in the paper, followed by the blinded name.

These changes make referring to particular origamis easier.

Figure 3d is difficult to interpret and appears redundant with Figure 3b, obscuring rather than clarifying the results.

We now moved Figure 3d to the Supplementary Information. It is Supplementary Figure 4 in the section which describes the M1 metric. It gives a visual illustration of how metric M1 detects off-target binding sites for the different triangle origami variants.

Conclusion

This paper addresses an important problem in DNA origami design and presents a novel framework with promising experimental validation. However, significant reorganization is needed to improve clarity, and experimental concerns should be addressed before publication. The authors should:

1. Move critical information from the supplement to the main text
2. Address the relationship with previous work more thoroughly
3. Clarify the experimental validation issues
4. Provide better details on methodology and implementation

With these improvements, this work could make a valuable contribution to the field of DNA nanotechnology.

Response to Reviewers - Reviewer #3

We thank the reviewer for their time analysing our manuscript. The reviewer comments have helped us clarify our text. We provide an annotated pdf with all the changes made to the paper but please find below a point-by-point response with review comments in blue, our response in black and any parts of the paper added or removed in green:

The manuscript by Ben Shirt-Ediss and co-workers explores (both computationally and experimentally) effects of off-target interaction minimization in DNA origami assembly. The authors defined four thermodynamic objective functions, or metric, (M1-M4) to characterized particular class of off-target binding and hypothesize that scaffolds with minimized metrics values yield better outcomes in DNA origami assembly process. The authors calculated Mi values for various scaffolds with sequences from biological vectors pool and also with Bruijn sequences. Assembly of two DNA origami constructs (rectangle and triangle) with three scaffold sequences with different metric for off-target bindings were evaluated experimentally using agarose gel electrophoresis and AFM. Experimental results mostly confirmed the initial hypothesis that origami designs with scaffolds the exhibit minimized off-target binding fold with better yields. The authors also used single molecule optical tweezers to characterize unfolding of DNA origami structures.

Overall, this is an example of very well designed and carefully executed research. I enjoyed reading the manuscript as writing is of high quality, and comprehensive computational and experimental data is presented very clearly. I appreciate that the authors openly discuss not only advantages but also limitations of their method. The presented outlook for further development is very reasonable and I am looking forward to follow-up studies. I have only minor comments related to the main text and the Supplementary Information (see below).

While the work is of high quality, I am not convinced that the scope makes it suitable for publication in multidisciplinary journal like Nature Communication. I would recommend publication in more specialized journal, e.g., Nucleic Acids Research.

We thank the reviewer for their careful reading. We have implemented the below minor changes.

Minor comments

Abstract: OT abbreviation is not defined in the abstract

Replaced with "optical tweezers".

Introduction: “..via hybridisation, melting and strand-displacement reactions in a decreasing temperature gradient”. The term “gradient” is not used correctly here, perhaps, the authors meant temperature ramp.

Now corrected to "ramp".

SI - It seems that reference are not properly displayed in several places, at least I see several ”?” instead of reference numbers.

There were two instances of ? in the SI; they are now proper references.

SI - It would be appropriate to use degree symbol “oC” whenever temperatures are mentioned. Currently, “o” is omitted at several places.

Instances in the Supplementary Material written as "C" have been corrected to "oC" (In the main paper, there were no instances of “C”, it was always written "oC")

Reviewer #4 (Remarks to the Author):

We thank the reviewer for their time analysing our manuscript. The reviewer comments have helped us clarify our text. We provide an annotated pdf with all the changes made to the paper but please find below a point-by-point response with review comments in blue, our response in black and any parts of the paper added or removed in green:

I co-reviewed this manuscript with one of the reviewers who provided the listed reports. This is part of the Nature Communications initiative to facilitate training in peer review and to provide appropriate recognition for Early Career Researchers who co-review manuscripts.

Thank you for co-reviewing.

Response to Reviewers (Round 2) - Reviewer #1

Review comments in blue. Response in black. Parts of the paper added or removed in green.

Reviewer 1:

Shirt-Ediss et al have edited their manuscript in response to my comments, and those of other reviewers. Most importantly, they have addressed my central criticism by including data for 2 new designs, which support the original story. Scientifically, therefore, the case is made for publication.

I am less convinced by the responses to my more minor comments. Sometimes an explanation is given, but something is not added to the text to explain the issue to other readers. See comments 2c,d,e, 2 (note, the numbering is off), 19, 22. In other cases, a explanation in the response seems a bit contradictory, even if the edited text is fine (see below). Although the response to these comments should probably not preclude publication, some may be worth reconsidering.

Minor points
1. How is the image in Fig 1A generated?

We added a reference to oxDNA.org in the Figure 1 caption:
"Three possible sequence variants are displayed for a triangle origami (rendered by oxDNA.org [REF])"

2. I note that the square images in the 6-helix bundle AFM images could be arranged more neatly, with the same dimensions and alignment.

We updated Supplementary Figure 42 to give the 6-helix bundle AFM images the same dimensions and alignment.

3. 2a - while the text added to the manscript is ok, the argument that it is "obviously wrong" to use worse case scenarios for M1 and M2 surely could equally be applied to M3 and M4?

We acknowledge the reviewer's point regarding the mathematical symmetry. However, our decision to use worst-case metrics for staples (M3,M4) versus totals for the scaffold (M1,M2) is based on the distinct biophysical failure modes of these components:

(1) The "Threshold" Nature of Staples (M3,M4): Staples function as discrete structural units. If a single staple possesses a very strong internal hairpin or staple-staple binding (e.g., large negative ΔG), it may remain folded at the annealing temperature and fail to bind the scaffold entirely. This results in a specific, localized defect in the origami and a "dead" staple.

- If we used a summation, a design with one fatal hairpin (e.g., -20 kcal/mol) would receive the same score as a design with ten harmless, transient hairpins (e.g., -2 kcal/mol each). The summation would mask the catastrophic outlier.
- By minimizing the worst case, we specifically target and eliminate these "fatal" outliers to ensure every staple is thermodynamically available to bind.

(2) The "Cumulative" Nature of the Scaffold (M1,M2): The scaffold is a single large molecule susceptible to kinetic trapping. Here, the danger is not a single binding event, but the cumulative friction of thousands of weak off-target interactions that create a rough energy landscape. Reducing the "worst" off-target site is insufficient if thousands of medium-strength sites remain. Therefore, the total summation is the physically relevant proxy for the global kinetic barrier.

We hope this clarifies that the choice was not arbitrary, but designed to address the specific thermodynamic risks associated with components versus the substrate.

4. One does not need to sample from nupack to get *all* the sequence averaged parameters, including loop closing terms - these can all be directly calculated from their definition in the supporting literature.

We do agree with the reviewer that the sequence-averaged parameters (including loop closing terms) could theoretically be derived from first principles by averaging the values in the supporting literature (e.g., SantaLucia et al., 2004).

However, we maintain our NUPACK Monte Carlo estimation approach for M1 and M2 for two specific reasons:

1) Complex Salt Corrections: The standard nearest-neighbour parameters in the literature are typically defined at 1 M NaCl. DNA origami experiments occur in Magnesium-rich buffers (e.g., 12.5 mM Mg2+). Converting literature parameters to experimental conditions requires specific salt correction algorithms (e.g., Owczarzy et al., 2008) which are non-linear and implementation-specific. NUPACK contains robust, pre-validated implementations of these salt corrections. By sampling NUPACK outputs, we automatically capture the exact salt-corrected free energies used in our experimental conditions without risking implementation errors by re-coding these complex corrections from scratch.

2) Internal Consistency: Since metrics M3 and M4 call NUPACK directly (because they need to use the fast NUPACK dynamic programming algorithms to calculate MFE structures), it is crucial that the approximated energy model for M1 and M2 operates on the same thermodynamic scale. Our sampling approach ensures that our "fast" model is a faithful surrogate approximation of the "full" NUPACK engine, capturing any specific handling of ensemble states (e.g., "No Stacking" settings) that might differ slightly if we implemented a fresh model solely from literature tables.

We regard both first-principles and Monte Carlo approaches as valid methodologies for parameter estimation. We chose the latter for the reasons outlined above.

5. It is still unclear to me what information the coloured boxes in Fig 1 convey beyond the fact that a system consists of sequences and scaffold. What do we learn from them? I don't think the authors use them to illustrate the idea that the same scaffold can use different sequences, for example. One ends up looking for meaning in the boxes that doesn't seem to be there.

The coloured boxes on Figure 1 have a fairly simple interpretation. The colour of the first box is a function of the scaffold sequence. The colour of the second box is a function of the complementary staple sequences. If the scaffold sequence is changed, the staple sequences change, and both colour boxes change colour (Figure 1a). If the scaffold sequence is rotated through the origami, the scaffold sequence stays the same and only

the colour of the staple sequences box changes. This possibility is not show in Figure 1a, but it is a feature in the software tool that accompanies our paper (see https://scaffoldselector.readthedocs.io/en/latest/select_rotation.html). The software tool not only changes the scaffold sequence, but it can also, if desired, rotate the scaffold sequence through an origami (rotating the scaffold sequence changes the M1, M3 and M4 scores but not M2). This is the origin of the two-colour box representation. Without scaffold rotation, a scaffold-staple set combination could be represented by a single distinct colour, which is perhaps what the reviewer is expecting. We hope this clarifies the issue.

6. Previous comment 16 - how does the (unreadable) dot parenthesis notation help "the idea that the figure conveys" (the profiles are similar)? If it doesn't, why include it?

We have removed the dot parenthesis notation from the x-axis of plots in Supplementary Figure 2, where the notation is crowded. Instead, we label the x-axis as “Base index along window”.

7. Previous comment 19 - I accept that the core function is not inhibited, but what value do these correlation plots provide? They do not directly contribute to the core function, unless I misunderstand.

The value of the correlation plots is to demonstrate the orthogonality of our four scoring metrics. Importantly, the plots show that the metrics are not linearly correlated (low Pearson coefficients). If two metrics had a persistently high correlation, it would signal that they are not deriving independent information about off-target interactions, and should therefore be combined to reduce the dimensionality of the optimisation problem. By demonstrating that M1 through M4 are statistically independent, we validate that they measure distinct thermodynamic phenomena (e.g., inter-staple binding vs. scaffold secondary structure). This independence confirms that a multi-objective framework is strictly necessary to capture the full landscape of off-target effects, as optimising one metric does not automatically optimise the others.

To incorporate the above points into the paper, we changed the sentence on p6 of paper
“This signified that the scoring metrics measured independent off-target binding phenomena, as desired”
to:
“This signified that the scoring metrics were orthogonal (derived independent information about off-target interactions)”.

8. Previous comment 22 - I couldn't find a definition of "Relative trace length" in the text itself.

“Relative trace length” is used in the caption of Supplementary Figure 47. We changed this caption to now define this concept:
“(a) Distribution of the length of the experimental traces. Shown is the fraction of experimental traces for each triangle variant that reached a relative trace length **(defined as trace length/scaffold length)** without breaking.”

Response to Reviewers (Round 2) - Reviewer #2

Review comments in blue. Response in black. Parts of the paper added or removed in green.

Reviewer 2:

The revisions are satisfactory. Aside from the following points, the manuscript is acceptable for publication.

1. Please define technical terms before they are presented in the main text (e.g., the Pareto set should be defined before it is used in the introduction, and the MCDM methods should be defined before they are used). There are additional places where the text could be simplified. For example, on page 6, the sentence about non-designed binding regions would be clearer if rephrased to directly state that random scaffolds are unlikely to contain repeated or complementary segments.

(1) The "Pareto set" concept is fully defined the first time it is used in the introduction. See this paragraph:
"The relative comparison of sequence variants takes place in objective space (Figure 1e) where a set (called the 'Pareto front') of optimal trade-off sequence variants becomes defined (black dots, see Supplementary Note 3). Sequence variants on the Pareto front are special in that they represent the set of optimal trade-offs for the optimisation problem: for Pareto variants, it is not possible to decrease the prevalence of one type of off-target reaction without (potentially) increasing the prevalence of other types of off-target reaction. A Pareto front exists because metrics are antagonistic and it is typically impossible for a sequence variant to minimise all metrics M1 to M4 to zero simultaneously."

(2) We improved the definition of MCDM when it is first used. Before the text was:
"Therefore, for the last step, we use a Multi-Criteria Decision Making (MCDM) method to help decide which single variant on the Pareto front is the most appropriate scaffold/staple sequence set to order for implementing the origami design (see Supplementary Note 3 for full details of SAW, KNEE and TOPSIS methods)."

And we changed the text to:
"Therefore, for the last step, we use a Multi-Criteria Decision Making (MCDM) method to help decide which single variant on the Pareto front is the most appropriate scaffold/staple sequence set to order for implementing the origami design. An MCDM method provides a mathematical framework to rank the trade-off variants on the Pareto front, allowing the identification of a single 'best compromise' solution, for example, by calculating which variant is geometrically closest to a theoretical 'utopia point' where all metrics are minimized. See Supplementary Note 3 for definitions of the SAW, KNEE and TOPSIS MCDM methods used."

(3) To simplify our mathematical explanation why random scaffolds perform well on p6:
"This performance can be attributed to the fact that a non-designed binding region on a random sequence is exponentially less likely to be matched by a complement as it grows in length."
We changed this sentence to the reviewer's suggestion:
"This performance can be attributed to the fact that random scaffolds are unlikely to contain repeated or complementary segments."

2. The authors have addressed previous concerns by adding experimental validation with a tetrahedron and 6-helix bundle. However, it should be noted that these wireframe designs are structurally similar to 2D shapes in terms of cross-section and folding difficulty. Future studies would benefit from including multilayer 3D origami structures, such as brick- or cube-like designs, of which there are numerous examples in the literature. This represents a more stringent test of the framework's predictive power for complex 3D assemblies.

We acknowledge that dense 3D multilayer origami structures (like bricks) could provide additional challenges to fold, mainly because inner regions need to fold before outer regions to stop steric hindrance from causing kinetic traps. However, we respectfully note that the 6-helix bundle used in our study is a solid cross-section object based on a hexagonal lattice, representing the fundamental packing unit of larger multilayer brick structures. Furthermore, the thermodynamic off-target traps our method identifies (e.g., strong scaffold secondary structure or staple co-folding) are intrinsic to the sequence mixture and compete with correct folding regardless of the final target geometry. Therefore, minimizing these off-target 'frictions' is likely still a prerequisite for successfully folding more complex, sterically challenged 3D shapes.

Our approach could effectively be adapted to include another “fold order” metric (M5) for on-target binding sites for densely packed 3D structures. However, we leave these types of investigations as tasks for future studies extending our work. Indeed, we feel our work leaves open many interesting possibilities for future research. The current paper provides initial experimental validation for the theoretical framework introduced.

Response to Reviewers (Round 2) - Reviewer #3

Review comments in blue. Response in black. Parts of the paper added or removed in green.

Reviewer 3:

I carefully read the revised manuscript, the reviewers 'comments and the authors ' responses. I had only minor comments for the original manuscript and all of those were satisfactory addressed.

However, as for the original submission, I am not convinced that this manuscript is suitable for publication in Nature Communications. While the work is of high quality, my hesitation to recommend it for the publication in Nature Communication comes from my, perhaps subjective, interpretation of the journal's aim and scope which states that "Papers published by the journal aim to represent important advances of significance to specialists within each field". I am having difficulty identifying "important advances of significance" in this work.

The work rather convincingly demonstrates that scaffolds with lower degree of off-target integrations improve yields in DO folding process. The authors also provide tools to compare the extend of off-target binding for different scaffold sequences. However, it is difficult for me to think of realistic scenario in which one would use those tools in practice for scaffold sequence optimization/selection*.

* I have been using DNA origami (DO) technique for over 15 years (folding origami since 2007) and while my research group focus is not in the field of structural DNA nanotechnology we utilizes the DO technique extensively. We care a lot about yields of DO assembly, still, minimization of off-target interaction is not a problem that we are concerned about in our DO design/fabrication/characterization pipeline. We (as probably vast majority of researchers using DO) use commercially available scaffolds. If our DO yields are not satisfactory, we optimize folding condition and, since recently, staple routing (Aksel et al).After such optimization, all our DNA origami designs (2D, 3D, twisted, bend, honeycomb and square lattice) fold well enough. This workflow is also very common in other labs, and I doubt that this work would change it, i.e., most of researchers will not optimize scaffold sequence as it is rather challenging from scaffold production point of view.

We thank the reviewer for his previous comments, which we have address in the last round. We would just like to clarify that, contrary to the perception that our method requires challenging de novo synthesis, our approach is specifically designed to optimize the usage of standard, commercially available vectors (such as the Lambda DNA used in our experiments). We provide fully documented open-source software that automatically identifies the optimal region within these common vectors to minimize off-target interactions; the user simply orders PCR primers to amplify this selected region, making implementation straightforward within a standard workflow.

Response to Reviewers - Reviewer #2

We thank the reviewer for their time reading our paper and for making helpful, detailed, comments, which have helped us improve it.

The reviewer requested *“A brief acknowledgment in the Discussion that validation on larger multilayer structures with significant fold-order constraints remains an open direction”*.

This has now been added.

The manuscript by Ben Shirt-Ediss and co-workers explores (both computationally and experimentally) effects of off-target interaction minimization in DNA origami assembly. The authors defined four thermodynamic objective functions, or metric, (M1-M4) to characterized particular class of off-target binding and hypothesize that scaffolds with minimized metrics values yield better outcomes in DNA origami assembly process. The authors calculated M_i values for various scaffolds with sequences from biological vectors pool and also with Bruijn sequences. Assembly of two DNA origami constructs (rectangle and triangle) with three scaffold sequences with different metric for off-target bindings were evaluated experimentally using agarose gel electrophoresis and AFM. Experimental results mostly confirmed the initial hypothesis that origami designs with scaffolds the exhibit minimized off-target binding fold with better yields. The authors also used single molecule optical tweezers to characterize unfolding of DNA origami structures.

Overall, this is an example of very well designed and carefully executed research. I enjoyed reading the manuscript as writing is of high quality, and comprehensive computational and experimental data is presented very clearly. I appreciate that the authors openly discuss not only advantages but also limitations of their method. The presented outlook for further development is very reasonable and I am looking forward to follow-up studies. I have only minor comments related to the main text and the Supplementary Information (see below).

While the work is of high quality, I am not convinced that the scope makes it suitable for publication in multidisciplinary journal like *Nature Communication*. I would recommend publication in more specialized journal, e.g., *Nucleic Acids Research*.

Minor comments

Abstract: OT abbreviation is not defined in the abstract

Introduction: "..*via hybridisation, melting and strand-displacement reactions in a decreasing temperature gradient*". The term "gradient" is not used correctly here, perhaps, the authors ment temperature ramp.

SI:

- It seems that reference are not properly displayed in several places, at least I see several "?" instead of reference numbers.
- It would be appropriate to use degree symbol "°C" whenever temperatures are mentioned. Currently, "°" is omitted at several places.
